# The Nuclear Receptor REV-ERBα Regulates *Fabp7* and Modulates Adult Hippocampal Neurogenesis

**DOI:** 10.1371/journal.pone.0099883

**Published:** 2014-06-16

**Authors:** Anna Schnell, Sylvie Chappuis, Isabelle Schmutz, Emanuele Brai, Jürgen A. Ripperger, Olivier Schaad, Hans Welzl, Patrick Descombes, Lavinia Alberi, Urs Albrecht

**Affiliations:** 1 Dept. of Biology, Unit of Biochemistry, University of Fribourg, Fribourg, Switzerland; 2 Dept. of Medicine, Unit of Anatomy, University of Fribourg, Fribourg, Switzerland; 3 NCCR frontiers in Genetics, University of Geneva, Geneva, Switzerland; 4 Dept. of Anatomy, University of Zürich, Zürich, Switzerland; University of Lübeck, Germany

## Abstract

The function of the nuclear receptor *Rev-erbα* (*Nr1d1*) in the brain is, apart from its role in the circadian clock mechanism, unknown. Therefore, we compared gene expression profiles in the brain between wild-type and *Rev-erbα* knock-out (KO) animals. We identified fatty acid binding protein 7 (*Fabp7, Blbp*) as a direct target of repression by REV-ERBα. Loss of *Rev-erbα* manifested in memory and mood related behavioral phenotypes and led to overexpression of *Fabp7* in various brain areas including the subgranular zone (SGZ) of the hippocampus, where neuronal progenitor cells (NPCs) can initiate adult neurogenesis. We found increased proliferation of hippocampal neurons and loss of its diurnal pattern in *Rev-erbα* KO mice. In vitro, proliferation and migration of glioblastoma cells were affected by manipulating either *Fabp7* expression or REV-ERBα activity. These results suggest an important role of *Rev-erbα* and *Fabp7* in adult neurogenesis, which may open new avenues for treatment of gliomas as well as neurological diseases such as depression and Alzheimer.

## Introduction

In mammals the circadian clock system regulates many aspects of systemic biology such as biochemistry, physiology and behavior with the suprachiasmatic nuclei (SCN) as the main coordinating entity to synchronize all cellular clocks in the body. At the cellular level, the circadian clockwork consists of interwoven positive and negative feedback loops, or ‘limbs’. The positive limb involves BMAL1/CLOCK heterodimers that bind to E-boxes located in the regulatory region of the *period* (*Per*) and *cryptochrome* (*Cry*) genes. CRY and PER proteins form oligomers that are transported from the cytoplasm to the nucleus, where they repress their own transcription by inhibiting BMAL1/CLOCK (negative limb). The positive and negative limbs are further interlaced as BMAL1/CLOCK also induces the expression of the nuclear receptor REV-ERBα (NR1D1), which represses the transcription of *Bmal1* via direct binding to a REV-ERBα response element (RORE) in the *Bmal1* promoter [1]. In addition to its action in the circadian clock mechanism, REV-ERBα also has strong regulatory functions in liver metabolism [2], [3] and drugs targeting it may have potential applications for treatment of metabolic syndrome [4]. However, the roles of REV-ERBα in the central nervous system remain unclear.

Components of the clock mechanism modulate neurogenesis. For example *Per2* regulates neural stem/progenitor cell proliferation in the adult hippocampus [5] while *Bmal1/Clock* seems to regulate neurogenic transcription factors such as Neuro D1 and differentiation of neuronal stem/progenitor cells in the subventricular zone (SVZ) of the lateral ventricle [6]. Furthermore, gene expression profiling revealed an increased expression of *Rev-erbα* in neural progenitor cells (NPCs) compared to immature neurons [7]. Outside of the central nervous system, in the skin, the clock appears to play a role in the regulation of stem cell differentiation [8], [9].

Adult neurogenesis is an important process, because it may replace lost or dysfunctional cells by generating new neurons via neural stem cells (NSCs) [10]. A dysfunction of this process may lead to neuropsychiatric diseases such as age-related cognitive decline [11] and depression (reviewed in [12]). Substantial generation of new neurons occurs mainly in two brain areas: the subventricular zone (SVZ) lining the lateral ventricles [13] and the subgranular zone (SGZ) of the hippocampal dentate gyrus (DG) [14]. Adult hippocampal neurogenesis in mammals is a sensitive process, which is affected by environmental stimuli, such as stress [15], [16], physical activity [17], sleep deprivation [18], enriched living conditions [19], and jet-lag [20], [21]. Such environmental changes directly affect the circadian clock [22], suggesting that the clock may be a mediator between environmental change and neurogenesis. This hypothesis is supported by the observation that neurogenesis fluctuates over the day [23]–[26] indicating that the circadian clock or components of it may influence neurogenesis.

Since REV-ERBα is strongly expressed in the brain [27] and in NPCs [7] we performed genome wide expression profiling in the SCN of wild-type and *Rev-erbα* KO mice. We found fatty acid binding protein 7 (FABP7), also termed brain lipid binding protein (BLBP), to be strongly up-regulated in *Rev-erbα* KO animals. FABP7 is a family member of the fatty acid binding protein family, which facilitates the solubility of hydrophobic long chain fatty acids. They function primarily in fatty acid uptake/transport [28], and have been widely implicated in cell growth and differentiation [29]. FABP7 is a well-known marker for NPCs [30] in neurogenic niches of the hippocampal SGZ [31] and in the forebrain SVZ [32]. It is expressed in type 2 and 3 NSCs and early transitory amplifying cells (TAPs) but not in late TAPs and neuroblasts [32]. Interestingly, *Fabp7* mRNA is expressed in a time of day dependent manner in hippocampal granule precursors in adult mice [33] and its localization and grade of polyadenylation are diurnal [34]. These observations implicate an involvement of circadian clock components in the regulation of *Fabp7* and adult neurogenesis.

In this study we show that *Fabp7* is a direct target gene of *Rev-erbα* and that both genes are involved in aspects of adult neurogenesis in mice.

## Methods

### Animal experiments

Animal handling and care was performed in accordance with the guidelines of the Schweizer Tierschutzgesetz (TSchG, SR455) and the declaration of Helsinki. The protocol was approved by the state veterinarian of the Canton of Fribourg. Suffering of animals was minimized by anesthesia that was induced at 4.5 to 5% isoflurane and lowered to 2–1.5% isoflurane mixed with oxygen (0.8l/min). *Rev-erbα^−/−^* knockout mice [27] were obtained from heterozygous *Rev-erbα* breeding pairs originally provided by Prof. U. Schibler, Geneva. Two to four month old animals were used for experiments and wild-type mice served as controls. Animals were kept under 12 h light and 12 h dark (LD 12∶12) with food and water *ad libitum*.

### Cell culture

NG 108-15, mouse neuroblastoma x rat glioblastoma cells [35] and U-251 MG, human malignant glioblastoma tumour [36] were used for in vitro experiments. Cells were maintained in Dulbecco's Modified Eagle Medium (DMEM), high glucose [4.5 g/l](Sigma 6429) containing 10% fetal calf serum (FCS) and 100 U/ml penicillin/streptomycin at 37°C in a humidified atmosphere containing 5% CO_2_. Sub-confluent cultures were split 1∶3 to 1∶6.

### Affymetrix oligonucleotide microarray hybridization

To obtain sufficient amounts of SCN, tissue of 18 male mice (3 months) were used per genotype. Dissection was performed at time point ZT 14. SCN of 6 animals were pooled to yield 3 samples of wild-type and *Rev-erbα^−/−^* mice each, and homogenized in RNA-Bee (AMS Biotechnology) using syringe and needle (Ø 0.19 mm). RNA extraction was performed with chloroform, followed by isorpropanol precipitation and wash with EtOH. For further purification RNA was precipitated again with 0.4 M NaOAc/0.2% SDS and extracted using phenol:chloroform:isoamylalcohol. RNA quality and integrity was checked by absorbance ratio A_260_/A_280_, on denaturing agarose gels and by using the Agilent 2100 Bioanalyzer. 5 µg of total RNA were employed for the synthesis of biotinylated cRNA and 17.5 µg of this cRNA were hybridized to Affymetrix Mouse Genome 430 2.0 array (according to the Affymetrix protocol). The signal intensities were analyzed using Partek Genomics suites (Partek, St. Louis, MI, USA) and Matlab (The MathWorks Inc., Natick, Massachusetts, USA) The data were normalized using RMA [37]. The selections were based on the fold-change intensities and p values (p<0.05). Genes for which the concordance in the pairwise comparisons exceeded the imposed threshold of 77% (seven out of nine comparisons) were considered as statistically significant and only transcripts whose accumulation had an average change of at least 1.5-fold were extracted (Tables S1 and S2).

### In situ hybridization

The *in situ* hybridization probe for *mFabp7* was cloned from cDNA corresponding to nucleotides 34–588 (accession number: NM_021272). Primer used for pCR II TOPO (Invitrogen) cloning are displayed in Table S1. Specimen preparation, ^35^S-rUTP labeled riboprobe synthesis and hybridization steps were performed as described earlier [38]. Quantification was performed by densitometric analysis of autoradiograph films (Amersham Hyperfilm MP) using the Quantity One 1-D analysis software (Biorad). Data from the region of interest was normalized by comparison with the signal intensities in an equal area of the lateral hypothalamus. Relative mRNA abundance was calculated by defining the maximal value of each experiment as 100%. Slides were further analyzed by dipping in NTB-2 emulsion and microscopy (Zeiss Axioplan 2). Silver grains were visualized with dark field illumination and tissue was visualized by counterstaining of nuclei with Hoechst-dye.

### Luciferase reporter assays and transfection

A 1.4 kb fragment of the mouse *Fabp7* promoter region (nucleotides -1′348 to the transcriptional start site, containing RORE at −934 and −257) was cloned into the pGL3 basic vector (Promega) using the primers indicated in Table S1. Deletion of the proximal RORE (nucleotide −257) was achieved by site directed mutagenesis using primers displayed in Table S1, which led to replacement of the proximal RORE (TGACCT) by nucleotides GATATC. Expression vectors for *Rev-erbα* (NM_145434) and *Rorα* (NM_013646) have been described [39] and an expression vector for β-galactosidase was used as control. Transfection and luciferase reporter assays were performed with NG108-15 cells (Neuroblastoma) according to [40]. Empty pGL3-vector and *Bmal1* promoter region cloned into pGL3 [39], were used as negative and positive controls, respectively. Real-time bioluminescence was monitored as described in [39] using a LumiCycle apparatus (Actimetrics).

### Chromatin immunoprecipitation (ChIP)

Hippocampal tissue was dissected using a mouse brain slicer (Zivic instruments). Freshly isolated tissue of two animals was combined for homogenization in 1% formaldehyde/1xPBS pH 7.4 and cross-linked for 5 min at RT. Nuclei and soluble chromatin fragments were obtained by ultracentrifugation through 1.8 M sucrose cushions and sonication according to [41]. Chromatin was precipitated with antibodies raised against REV-ERBα [42] and co-immunoprecipitated DNA was quantified with TaqMan real-time PCR using the primers and probes described in Table S1. ChIP data was normalized against corresponding input data and results were presented as percent of input.

### Quantitative Real-time PCR (qRT-PCR)

Total RNA was extracted and purified from snap frozen brain tissue using RNeasy kit (Qiagen) with on column DNAse digestion. RNA from cultured cells was extracted using RNA-Bee (AMS Biotechnology), purified by phenol:chloroform extraction and ethanol precipitation. cDNA was synthesized with SuperScript II (Invitrogen) and random priming. SYBR green fluorescence-based real-time PCR was performed for RNA quantification (KAPA SYBR FAST Universal, KAPA Biosystems, RotorGene 6000, Corbett Research). All RNA samples were normalized to *Gapdh*. Primers are listed in Table S3.

### Western blot analysis

Using RIPA buffer (50 mM Tris-HCl pH 7.4, 150 mM NaCl, 1 mM EDTA, 0.1% SDS, 1% Triton X-100, 0.5% sodium deoxycholate containing protease and phosphatase inhibitors), protein of cultured cells and brain tissue was extracted. Proteins were separated on 12.5% SDS-PAGE and transferred to nitrocellulose (Protran BA 83, 0.2 µm pores, GE healthcare). Primary antibodies were incubated over night at 4°C, Anti-rabbit FABP7 1∶250 (Abcam ab27171), Anti-rabbit actin 1∶5000 (Sigma, A5060) and Anti-BMAL1 1∶1000 [42]. Detection of the immune complexes was performed using Western Bright Quantum system (Advansta) and quantification was done with the Quantity One analysis software (BioRad). Actin was used for normalization and relative protein levels were calculated by defining maximal protein levels as 1.

### Behavioral studies

*Porsolt Forced swim tests* were performed using a cylindrical tank (35 cm height, 25 cm diameter) filled with water to a height of 20 cm. The water temperature was maintained at 27±2°C. An initial period of 2 min was given for habituation, then immobility time was recorded during 4 min using a stopwatch. Mice were considered immobile, when no obvious limb movements were observed and the floating body did not move actively through the water. After a total session of 6 min, mice were warmed up on a heating pad, and then placed back into their home cage. Mice were tested the same time of day (ZT 6 and ZT 18) at three subsequent days and mean values were plotted as cumulative immobility time in seconds.

For *Tail suspension tests* mice were suspended individually from the tail, fixed to a cord hanging in a box (36.5 cm high, 30.5×30.5 cm^2^) (according to the EMPRESS standard operating procedure http://empress.har.mrc.ac.uk). Animals were judged to be immobile when not agitating and not attempting to escape. Immobility was recorded during 6 min with a stopwatch. Tests were repeated for three subsequent days at the same time point (ZT 6 and ZT 18) and mean values were plotted as cumulative immobility time in seconds.

*Prepulse inhibition (PPI) tests* were carried out with acoustic stimuli. Mice were tested in a startle chamber (SR-lab System, San Diego instruments) positioned within a sound-proof cabinet in a sound-attenuating room according to standard methodology [43]. A constant background white noise of 64 dB was presented throughout the test. To measure prepulse inhibition, mice were presented with a 68, 72, 76, 80 and 84 dB prepulse (for 20 ms) followed by a 120 dB pulse (for 40 ms in length) 100 ms later. The percentage PPI of the startle response was calculated using the following formula: 100-[(SRPP/SR)x100]. SR denotes the startle response to the pulse stimulus, whereas SRPP denotes the startle response to the pulse with prepulse stimulus.

*Elevated O-maze test* was used to test anxiety, which affects mood-related behaviors. The relationship between curiosity/exploration and fear/hiding in a protected area is investigated. The elevated O-maze consisted of an elevated (42 cm above the floor) annular runway (outer diameter was 46 cm with 5.5 cm in with) divided into 4 sectors. The two 90° closed sectors were protected by 11 cm high inner and outer walls, while the remaining two open sectors were unprotected. Animals were released at the interface of the closed and open area and recorded for 5 min using a video camera. Number of entries and time spent in the open sector were counted. In order to avoid habituation to the maze, mice were tested once for a total session of 5 min.

*Y-maze spontaneous alternations test* was performed to test the working memory of mice using a Y-shaped maze with three plastic arms (height: 12.7 length: 38.3, width: 7.6 cm) at 120° angles. After introduction in the middle of the maze, mice were allowed to freely explore the three arms for 5 minutes. Sessions were videotaped and the sequential entries into each arm (A, B, C) were noted. An arm entry was scored when all four limbs of the animal were within an arm. Each set of three consecutive choices where no repeated entries occurred (counting also overlapping triplets) was scored as alternation. The Y-maze score was calculated as follows [number of alternations/(number of total entries-2)*100], a Y-maze score of 50% indicates random selection of arm entries. The maze was cleaned with 70% ethanol after each test. Tests were performed during the resting phase of mice between ZT 4 to ZT 6.

*Spatial object recognition (SOR)* tasks were performed in an arena (30×30×30 cm) with two objects, a plastic square (6.5×2.5×8.5 cm) and a metal cylinder (h: 9 cm, r: 2.2 cm). The bottom plate of the arena was decorated on one side with black and white stripes as a spatial cue. Mice were habituated to the empty arena for 10 min, subsequently the arena was cleaned with 70% ethanol and two objects were placed in the arena at opposite corners (upper left and lower right). The mice were introduced in the center of the arena and allowed to explore the arena for 10 min (object training). Object training was repeated on three consecutive days (24-h intervals) for 10 min each. Twenty-four hours after the third training one object was displaced to a new location (displaced object, DO) while the other object was not moved (non-displaced object, NOD) and the mice were allowed to explore the new situation during 10 min. The identity of the DO (plastic square or metal cylinder) was balanced between groups. The third training session and test session were videotaped. The response to spatial change was assessed by calculating the percentage of time spent exploring the DO vs. NOD. Exploration was scored when mice were facing and sniffing the objects within very close proximity and/or touching them.

### Immunohistochemistry

Animals used for immunohistochemistry were sacrificed at ZT 6. Perfused brains were cryoprotected and sectioned (40 µm, coronal) using a cryostat. Sections chosen for staining were placed in 24-well plates (up to 4 sections of one sample per well), washed 3 times in 1xTBS and twice in 2xSSC pH 7.0 (0.3 M NaCl/0.03 M tri-Na-citrate). Antigen retrieval was performed with 50% formamide/2×SSC by heating to 65°C for 50 min. Then, sections were washed twice in 2xSSC and 3 times in 1xTBS pH 7.5 (0.1 M Tris/0.15 M NaCl), before blocking them for 1 h in 10% fetal bovine serum (FBS)/0.1% Triton X-100/1xTBS at room temperature (RT). Directly after the blocking step, primary antibodies (DCX [Santa Cruz, SC8066], NeuN [Millipore, MAB377], FABP7 [abcam ab27171]) diluted in 1% FBS/0.1% Triton X-100/1xTBS were added to the sections and incubated overnight at 4°C. The next day sections were washed 3 times in 1xTBS and incubated with the appropriate fluorescent secondary antibodies diluted 1∶500 in 1% FBS/0.1% Triton X-100/1×TBS for 3 h at RT (Dk-Anti-mouse Cy5 [715-605-150], Dk-Anti-rabbit Cy2 [711-545-152], Dk-Anti-rabbit Cy3 [711-165-152], Dk-Anti-goat Cy3 [705-165-147], all from Jackson Immuno Research). After 3 washes with 1xTBS, nuclei were counterstained with DAPI for 10 min. Finally the tissue sections were washed again twice in 1xTBS and mounted on glass microscope slides. Slides were stored horizontally for at least one day at 4°C to allow the mounting medium to solidify. Fluorescent images were taken by using a confocal microscope (Leica TCS SP5), equipped with objectives 10x, 20x, and 40x, and an inverted DMI6000 stand with motorized stage. Images were taken with a resolution of 1024×1024, scan speed 400 Hz and Z-stack of 1.5 µm through the whole section with frame average 3. Images were processed with LAS AS software from LEICA.

### Assessment of cell proliferation and neurogenesis

Mice aged 6–12 weeks were used for the assessment of neurogenesis. To assess the total amount of newborn cells in the adult dentate gyrus, bromodesoxyuridin (BrdU) was administered by intraperitoneal injection (ZT6) at 100 mg/kg body weight and the mice (3 per genotype) were sacrificed 4 days later at ZT6. For the diurnal evaluation of proliferation, mice received a single dose of BrdU (100 mg/kg body weight) for 10 hour labeling. The injection schedule was as follows: injection at ZT 1 and perfusion at ZT 11 for light phase labeling; injection at ZT 13 and perfusion at ZT 23 for dark phase labeling (4 mice per genotype and time point). The tissue was fixed by cardiovascular perfusion, cryopreserved and sections of 40 µm were cut using a cryostat. For immunohistochemical detection of BrdU streptavidin-biotin detection was chosen. Free-floating sections were incubated in 1 M HCl on ice for 10 min, then in 2 M HCl at RT for 10 min and finally in 2 M HCl at 37°C for 20 min. Incubation in 0.1 M boric acid at pH 8.5 for 12 min was performed for neutralization. Sections were blocked for 1 h in 10% FBS/0.1% Triton X-100/1×TBS at RT, followed by specific blocking of streptavidin and biotin binding sites in the tissue (Streptavidin-Biotin blocking kit Vector labs). Primary antibodies diluted in 1% FBS/0.1% Triton X-100/1×TBS were added to the sections and incubated overnight at 4°C. Antibodies were Anti-DCX (abcam ab18723), Anti-BrdU [BU1/75 (ICR1)] (abcam ab6326) and Anti-NeuN (Millipore MAB377). Secondary antibodies were biotinylated Anti-rat (Vector Laboratories BA9400), Anti-mouse Cy5 and Anti-rabbit Cy3 (Jackson Immuno Research 715-605-150 and 711-165-152) for 3 h at RT and subsequently Streptavidin-FITC conjugate (Vector Laboratories SA5001) 2 h at RT. Mounted tissue sections were analyzed with a confocal microscope (Leica TCS SP5). Fluorescent images covering the DG region were taken with 40× magnification and Z-stack of 1.5 µm through the entire coronal section with frame average 3. Images were processed with LAS AS software from LEICA. To estimate the number of immunolabelled BrdU^+^ cells in the dentate gyrus (DG), systematic random sampling of every sixth 40-µm coronal section along the rostro-caudal axis of the DG (−1.06 mm to −3.80 mm from bregma) was chosen and performed according to [5]. Immunopositive cells were counted and the total amount of cells per DG was calculated by multiplying the results by six (because every sixth section had been used).

### Knockdown of Fabp7 by siRNA

SiRNA-mediated gene knockdown was achieved by using Lipofectamine RNAiMAX transfection kit (Invitrogen). U-251 MG cells plated to 6-well plates and grown to 30-50% confluence were transfected with 10 nM Stealth siRNA duplexes (Invitrogen): FABP7HSS103516, FABP7HSS103517, FABP7HSS103518 and siRNA negative control medium GC. Knockdown efficiency was assessed 72 h post-transfection by western blotting and real-time PCR.

### SR8278 (REV-ERBα antagonist) treatment

25 mM SR8278 (Sigma) stock solution in DMSO was prepared. Confluent cells were incubated during 24 h in presence of 10 µM SR8278, if not otherwise stated. Equal volumes of DMSO were used as control treatment.

### Cell migration assay

Experiments were carried out with 24-well plates and polycarbonate Trans-well membrane inserts containing 8 µm pores (Corning, 3422). 72 h after siRNA mediated gene knockdown and 18 h after addition of 10 µM SR8278 (antagonist of REV-ERBα) in DMSO (equal volumes of DMSO were used as control treatment), U-251 MG cells were removed from the plate using 0.1% trypsin in 1xPBS and counted. 20’000 cells, in DMEM without FCS, were plated to trans-well inserts and put in the receiver-wells. DMEM containing 10% FCS in the receiver-well was used as attractant. To allow migration, cells were incubated for 6 h in a CO_2_ incubator. A Q-tip was used to remove non-migrated cells from the upper side of the membrane, whereas migrated cells on the lower side of the membrane were fixed and stained for 10 min in 0.5% crystal violet/25% methanol. The number of migrated cells was determined by counting them in three random big squares of a Neubauer chamber and the results were displayed as percent of migrated cells of the total amount of cells plated per trans-well (3 mm^2^). Experiments were performed in duplicates and repeated least three times. Representative pictures of migrated cells were taken with a Zeiss Axioplan 2 microscope.

### Proliferation study

The Luna automated cell counter (Logos Biosystems) was used to assess proliferation of U-251 MG cells. Experiments were carried out 72 h post transfection and 18 h after treatment with 10 µM SR8278. Cells were detached using 0.1% trypsin in 1xPBS (2 min at 37°C), resuspended in growth medium and mixed with an equal volume of trypan blue stain (0.4% in 1xPBS). 10 µl of stained cell suspension was used per cell counting chamber, samples were counted twice and experiments were performed four times. Results were displayed as total cell number per well, since the ratio of living and dead cells did not vary between samples.

### Statistical analysis

Statistical evaluation of all experiments was performed using GraphPad Prism4 software. Depending on the type of data, either unpaired t-test, 1- or 2-way ANOVA with Bonferroni post-test was performed. Values were considered significantly different with p<0.05 (*), p<0.01 (**), or p<0.001 (***).

## Results

### Genome wide analysis reveals an increase of *Fabp7* in the SCN of *Rev-erbα* KO mice

**Figure 1:**
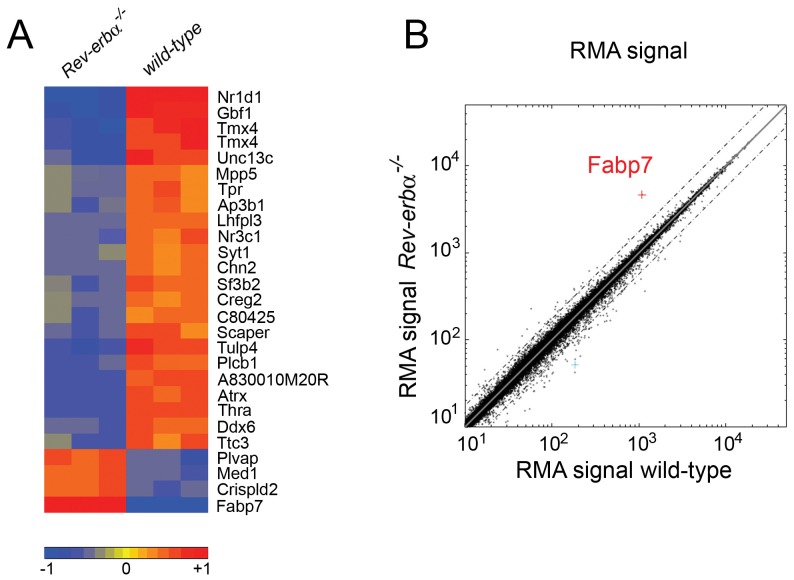
Genome wide expression profiling of wild-type and *Rev-erbα* ^−/−^ tissue from suprachiasmatic nuclei (SCN). (**A**) The strongest up- and down-regulated genes are displayed with red color marking the up-regulated genes and blue the down-regulated genes. The heat map is a selection of genes from the array based on p<0.01 (t-test) and an absolute fold change greater than 1.75. The color code is based on the log2 value of the fold change of the RMA values. The three columns per genotype represent the individual experiments (n = 3). (**B**) Plot of the RMA signals from wild-type versus *Rev-erbα* ^−/−^ SCN reveals *Fabp7* as the most up-regulated gene in *Rev-erbα* ^−/−^ mice (dotted lines represent 2 fold changes).

In order to detect differences in gene expression in brains of wild- type versus *Rev-erbα* KO mice we performed a microarray analysis. We focused our analysis on the SCN, because REV-ERBα is a component of the circadian clock of which the pacemaker resides in the SCN [44]. In order to identify *Rev-erbα* regulated genes, we collected tissue 2 hours after the beginning of the activity phase at zeitgeber time (ZT) 14, which is 2–4 hours after maximal mRNA expression of *Rev-erbα* [27]. Using Affimetrix whole genome arrays we identified a number of differentially expressed genes between the two genotypes (up-regulated genes Table S1, down-regulated genes Table S2). The strongest differences in gene expression are summarized in Figure 1. The list of up-regulated genes includes *Bmal1* (*Arntl*) and *Npas2*, two clock components that are directly regulated by REV-ERBα [27], [45]. We focused on genes that were up-regulated in *Rev-erbα* KO mice (red, lower part in Fig. 1A), because REV-ERBα acts as a repressor binding to RORE elements in the promoter of target genes. Lack of *Rev-erbα* will therefore lead to up-regulation of direct target genes. Plotting the RMA (Robust Multi-array Analysis) signals [37] from *Rev-erbα* KO versus wild-type mice clearly identified *Fabp7* as the most up-regulated (4.5-fold) gene in the SCN of *Rev-erbα* KO animals (Fig. 1B).

### *Fabp7* is over-expressed in different brain regions of *Rev-erbα* KO mice

**Figure 2:**
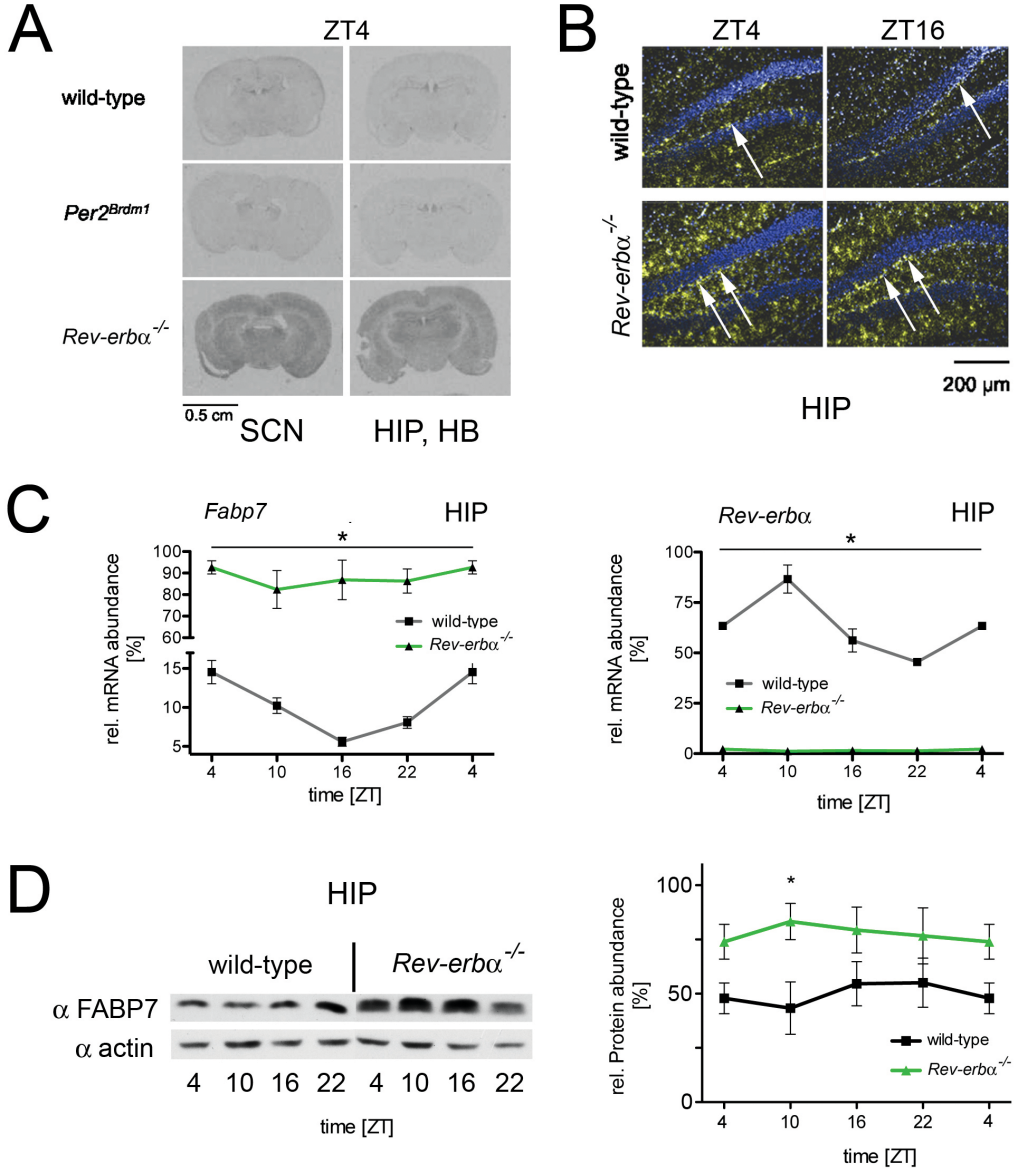
Expression profile of *Fabp7* mRNA and protein in brain tissue. (**A**) In situ hybridization on coronal brain sections of wild-type, *Per2^Brdm1^*, and *Rev-erbα^−/−^* mice at ZT4. The sections in the left column contain the SCN, the sections in right column the hippocampus (HIP) and habenula (HB). (**B**) The panel shows dark-field microscopy of the hippocampus (HIP) in the dentate gyrus region. Blue represents Hoechst-dye stained cell nuclei and the yellow signal represents the hybridization signal detecting *Fabp7* mRNA. (**C**) Quantification of *Fabp7* mRNA in the hippocampus of wild-type (black) and *Rev-erbα^−/−^* mice (green) over the period of 24 hours (left panel). The right panel depicts the *Rev-erbα* mRNA in the hippocampus of wild-type (black) and *Rev-erbα^−/−^* mice (green). 2-way ANOVA reveals a significant difference between wild-type and *Rev-erbα^−/−^* mice (n = 3, p<0.05, mean ± SEM). (**D**) The left panel shows a Western blot illustrating FABP7 protein levels in the hippocampus area of wild-type and *Rev-erbα^−/−^* mice. The right panel illustrates the quantification of FABP7 signal. 2-way ANOVA reveals a significant difference between wild-type and *Rev-erbα^−/−^* mice (n = 3, p<0.05, mean ± SEM).

As a next step we verified the increased expression of *Fabp7* in *Rev-erbα* KO mice using in situ hybridization, quantitative RT-PCR (qRT-PCR) and further extended the validation at the protein level by Western blotting. In situ hybridization experiments performed on brain slices of wild-type, *Per2^Brdm1^* mutant and *Rev-erbα* KO animals revealed increased expression of *Fabp7* mRNA in *Rev-erbα* KO mice. This expression was increased not only in the SCN, but also throughout various brain regions, including the hippocampus (HIP), the habenula (HB) (Figs. 2A and S1) and the cortex (CX) (Fig. S1), which are known sites of *Fabp7* expression [33]. In wild-type and *Per2^Brdm1^* mutant mice, *Fabp7* mRNA displayed a shallow diurnal pattern of expression (black and red lines, respectively) in the SCN and HB, whereas in *Rev-erbα* KO animals, this expression was elevated at all time points (green line, Fig. S1). More detailed analysis of the in situ hybridization experiment revealed that in *Rev-erbα* KO mice, *Fabp7* expression appeared to be elevated in the molecular layer (Fig. 2B) and the SGZ of the hippocampus (arrows, Fig. 2B). Next we quantified *Fabp7* by qRT-PCR in hippocampus and found it to be expressed in a phase consistent with the repression of its expression by REV-ERBα (Fig. 2C). Similar to the increase in mRNA expression of *Fabp7* Western blot analysis on hippocampal extracts from wild-type and *Rev-erbα* KO mice revealed elevated levels of FABP7 protein in the brain of *Rev-erbα* KO mice (Fig. 2D), suggesting that *Fabp7* is a target gene of REV-ERBα.

### REV-ERBα regulates *Fabp7* expression in vitro and in vivo

**Figure 3:**
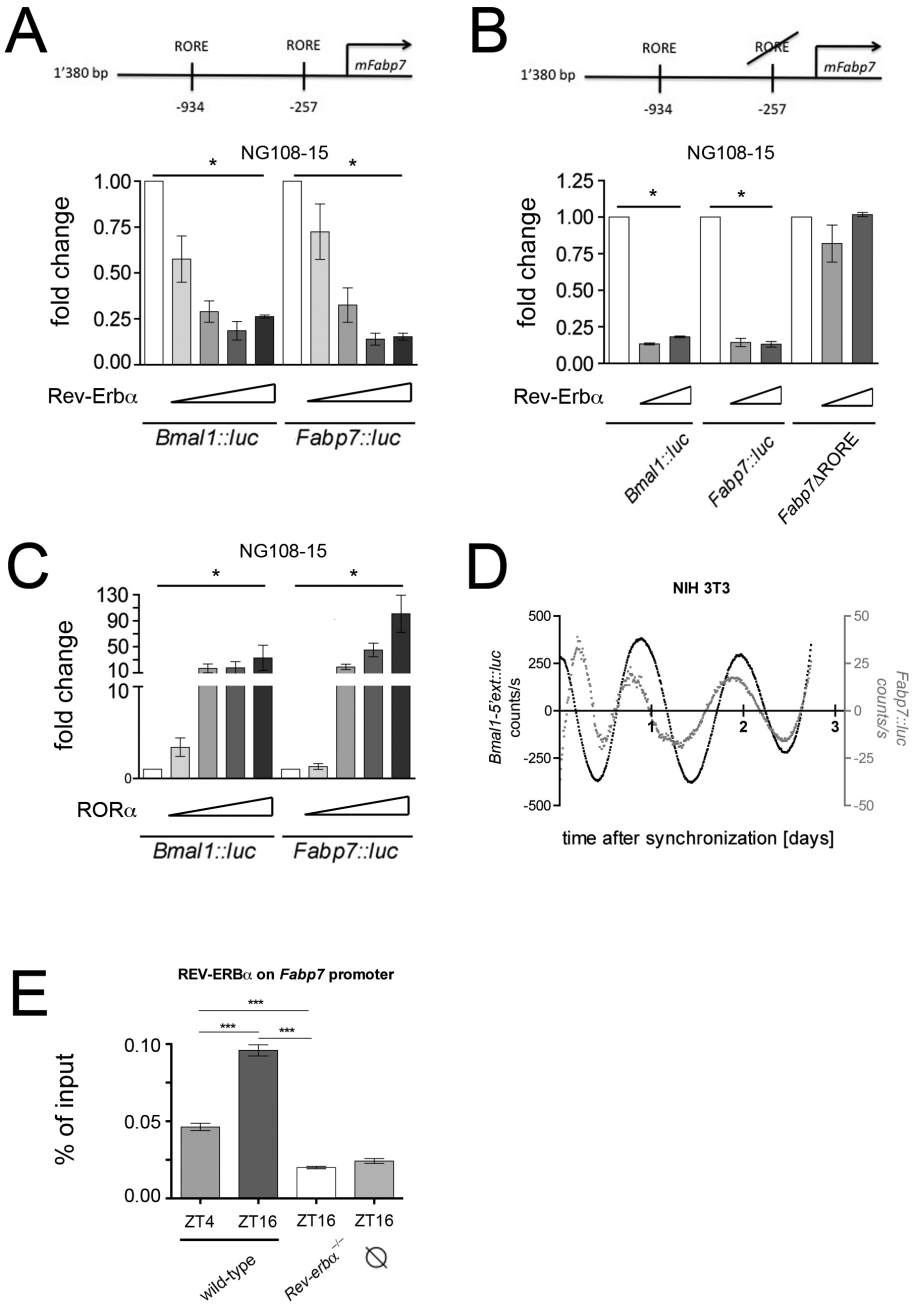
Molecular regulation of the *Fabp7* promoter. (**A**) The top panel depicts the murine *Fabp7* (*mFabp7*) promoter with its two REV-ERBα response elements (ROREs). Transactivation experiments in NG108-15 neurobalstoma cells show a repression potential of REV-ERBα that is similar for both the *Bmal1::luc* and *Fabp7::luc* reporter constructs (n = 3, *p<0.05, mean ± SD). (**B**) Deletion of the RORE element 257 nucleotides upstream of the transcription start site of *Fabp7* (*Fabp7Δ*RORE) abolishes the repression by REV-ERBα (n = 3, *p<0.05, mean ± SD). (**C**) RORα activates the *Fabp7::luc* reporter in a similar fashion as the *Bmal1::luc* reporter (n = 3, *p<0.05, mean ± SD). (**D**) Real-time monitoring of NIH 3T3 cells transfected with the *Bmal1::luc* and *Fabp7::luc* reporters, respectively. (**E**) Chromatin immunoprecipitation (ChIP) reveals time of day dependent binding of REV-ERBα on the *Fabp7* promoter in hippocampal tissue (n = 4, ***p<0.001, mean ± SEM, one-way ANOVA). ∅ denotes background binding of REV-ERBα at the unrelated *Fgf21* promoter.

Expression analysis suggested that *Fabp7* may be directly regulated by REV-ERBα. To test this hypothesis we performed transactivation experiments using a part of the *Fabp7* promoter fused to luciferase (*Fabp7::luc*) as a reporter and transfected this construct into the neuroblastoma cell line NG108-15. We found that REV-ERBαrepressed the activity of the *Fabp7* promoter in a dose dependent manner comparable to the known REV-ERBα mediated repression of the *Bmal1* promoter (Fig. 3A). Deletion of the proximal REV-ERBα binding element (RORE) on the *Fabp7* promoter (−257 nt upstream of the transcription initiation site) abolished REV-ERBα mediated repression (Fig. 3B). Interestingly, the positive acting counterpart of REV-ERBα, the retinoic acid related orphan receptor alpha (RORα), which also binds to ROREs, activated the *Fabp7* promoter in a similar fashion as it activates *Bmal1* (Fig. 3C). These results indicate that *Fabp7* is regulated by the nuclear receptors REV-ERBα and RORα. Since these two nuclear receptors are components of the circadian clock, we tested whether *Fabp7* is activated in a time dependent fashion. First, we verified that *Fabp7* is regulated by a similar mechanism in NIH 3T3 fibroblasts (Fig. S2). Thereafter, we monitored cyclic expression of *Fabp7::luc* after synchronization of cells with dexamethasone (Fig. 3D). Our experiments indicated a time dependent regulation of the *Fabp7* promoter in vitro in phase with *Bmal1*.

In a next step, we tested whether REV-ERBα binds to the *Fabp7* promoter in vivo by performing chromatin immunoprecipitation (ChIP) using chromatin prepared from the hippocampal area. REV-ERBα bound to the *Fabp7* promoter in a time dependent fashion and this binding was absent in *Rev-erbα* KO mice (Fig. 3E). This suggests that our observations made in cell cultures most likely also apply in vivo.

### *Rev-erbα* KO mice show alterations in mood-related behaviors and hippocampus-dependent cognitive performance

**Figure 4:**
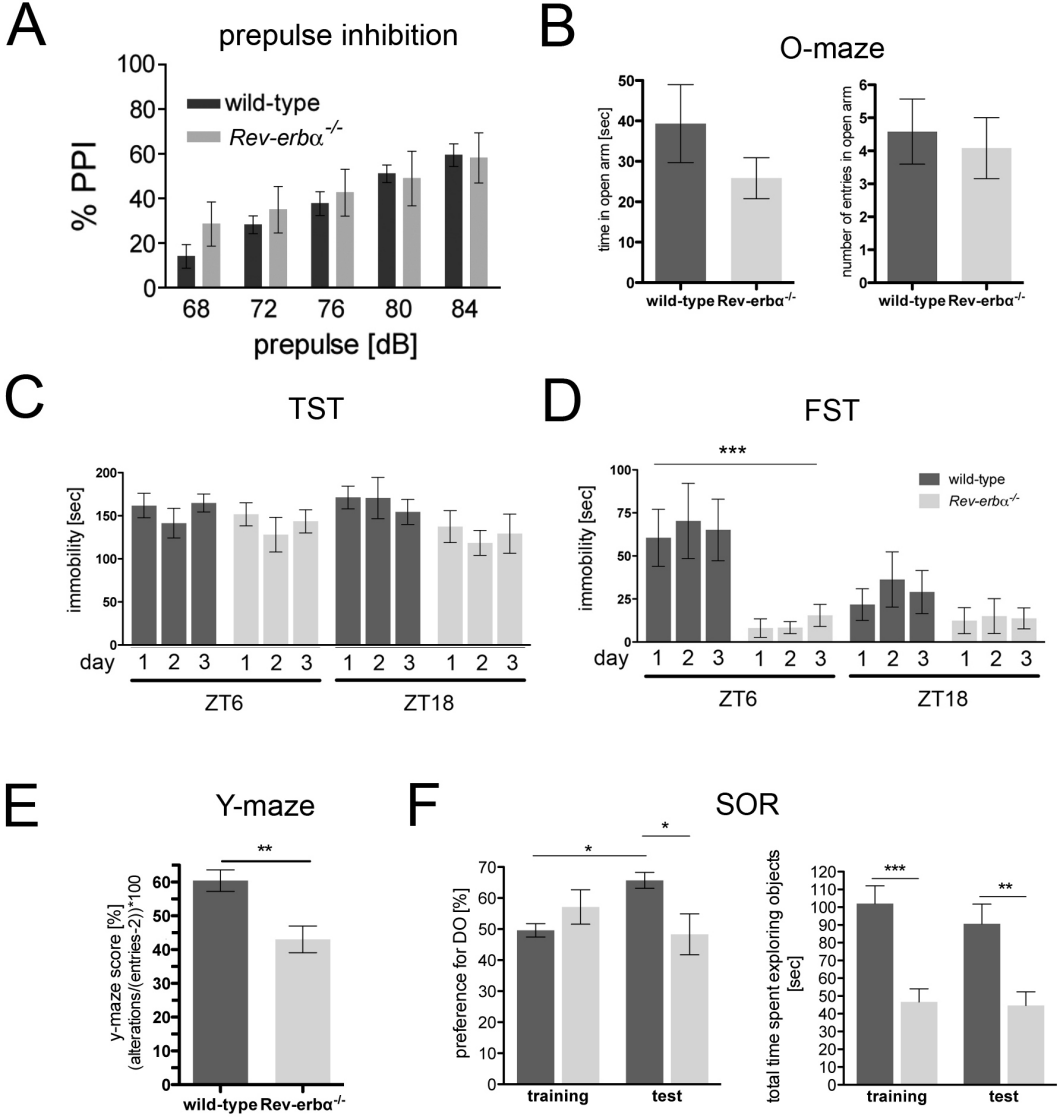
Mood-related behavior and hippocampus-dependent cognitive performance is altered in *Rev-erbα^−/−^* mice. (**A**) Mice were subjected to prepulse inhibition (PPI) during the light phase. Startle response after a prepulse at 68, 72, 76, 80 and 84 dB, followed by a pulse at 120 dB represented as percentage of PPI with 100% as the first absolute startle values. Wild-type and *Rev-erbα^−/−^* mice display a comparable amount of PPI, which is a measure related to schizophrenia (mean ± SEM, n = 6). (**B**) Mice were tested at ZT0-2 in the anxiety related elevated O-maze test and no significant differences between the two genotypes were observed (mean ± SEM, n = 12, 2-way ANOVA). (**C**) Mice were subjected to the tail suspension test (TST) at ZT6 and ZT18. No differences between the genotypes could be observed (mean ± SEM, n = 6, 2-way ANOVA). (**D**) Mice were subjected to the forced swim test (FST) at ZT6 and ZT18 for 3 consecutive days. *Rev-erbα^−/−^* mice were significantly less immobile compared to wild-type animals at ZT6 (mean ± SEM, n = 10, ***p<0.001, 2-way ANOVA). (**E**) Short term spatial memory was assessed between ZT4-6 using the Y-maze test (mean ± SEM, Student's t-test, **p<0.01, n = 12). (**F**) Long term spatial memory was assessed between ZT4-6 using the spatial object recognition test (SOR). The left panel shows the preference for the displaced object (DO) ± SEM (2-way ANOVA, p<0.05, n = 10). The right panel shows the total time exploring objects in general (mean ± SEM, 2-way ANOVA, **p<0.01, ***p<0.001, n = 10).

Next, we performed behavioral tests comparing wild-type and *Rev-erbα* KO mice. *Fabp7* maps to a quantitative trait locus for a schizophrenia endophenotype [46] and therefore we employed a prepulse inhibition (PPI) test, which is used as a measure for schizophrenia. However, we found no difference between wild-type and *Rev-erbα* KO mice (Fig. 4A), which overexpress *Fabp7*, in contrast to animals lacking *Fabp7*, which displayed a reduced response in the PPI test [46]. In order to test anxiety related behavior we used the elevated O-maze test during the light phase. Both genotypes spent the same amount of time in the open area (Fig.4B, left panel) and also the number of entries into the open area was similar (Fig. 4B, right panel), indicating no significant differences in anxiety. Next, we performed despair-based behavioral tests that detect differences in mood-related behavior such as mania and depression. The two genotypes did not differ in their behavior in the tail suspension test (TST) at ZT6 as well as at ZT18 (Fig. 4C). The data are shown over 3 consecutive days to illustrate no changes due to learning or adaptation. A more sensitive mood-related behavioral test, the forced swim test (FST) revealed a tendency of wild-type animals towards higher immobility at ZT6 compared to ZT18 (Fig. 4D). This is consistent with our previous observations [47]. Interestingly, *Rev-erbα* KO mice showed significantly reduced immobility compared to wild-type animals at ZT6 (Fig. 4D), although the total locomotor activity levels are similar to wild-type animals [27]. In the literature reduced immobility is often associated with ‘mania-like’ behavior, however, it may also reflect a deficit in learning to adapt to a hopeless situation [48], [49]. Therefore, we tested both genotypes in memory related tests. Spontaneous alterations in a Y-maze are considered a test of short term or working memory. Mice tend to avoid an arm they just have visited and alternate their entries among the arms, so re-entry into an arm just visited suggests memory impairment [50]. In the Y-maze task, *Rev-erbα* KO mice showed reduced spontaneous alterations between the arms compared to wild-type animals (Fig. 4E), suggesting a deficit in working and short-term memory. To assess long-term memory we employed the spatial object recognition test (SOR), which relies on the innate propensity of mice to explore their environment and recall where objects are located [51]. After training the mice to learn the position of objects, one object was moved 24 hours later. Wild-type mice will recall the position of the nondisplaced object (NDO) and the exploration of the displaced object (DO) will be favored. During the training session neither wild-type nor the *Rev-erbα* KO animals showed a preference for either object, but *Rev-erbα* KO mice showed less preference for the DO than wild-type mice when tested 24 hours later (Fig. 4F, left panel). However, it appeared that *Rev-erbα* KO animals were in general less explorative than wild-type animals (Fig. 4F, right panel). The SOR test suggests long-term memory deficits of *Rev-erbα* KO mice as previously observed [52]. Taken together the results indicate that *Rev-erbα* KO animals display impaired hippocampal functions regarding mood-related behavior and memory. We do not know, however, whether these two behavioral phenotypes are functionally related, since *Rev-erbα* may be responsible for the regulation of several transcriptional events in the brain as evidenced in figure 1A. In addition to *Fabp7* up-regulation many other genes including the glucocorticoid receptor (*Nr3c1*) are down-regulated in *Rev-erbα* KO mice and therefore it is very likely that the behavioral phenotypes are the result of changes in more than one transcriptional network.

### Neurogenesis and FABP7 protein expression are increased in the dentate gyrus of *Rev-erbα* KO mice

Neurogenesis-deficient mice exhibit increased immobility in the FST thus indicating a direct role of adult neurogenesis in depressive illness [53]. Therefore we hypothesized that *Rev-erbα* KO animals, which show decreased immobility in the FST (Fig. 4D), may display increased neurogenesis.

**Figure 5:**
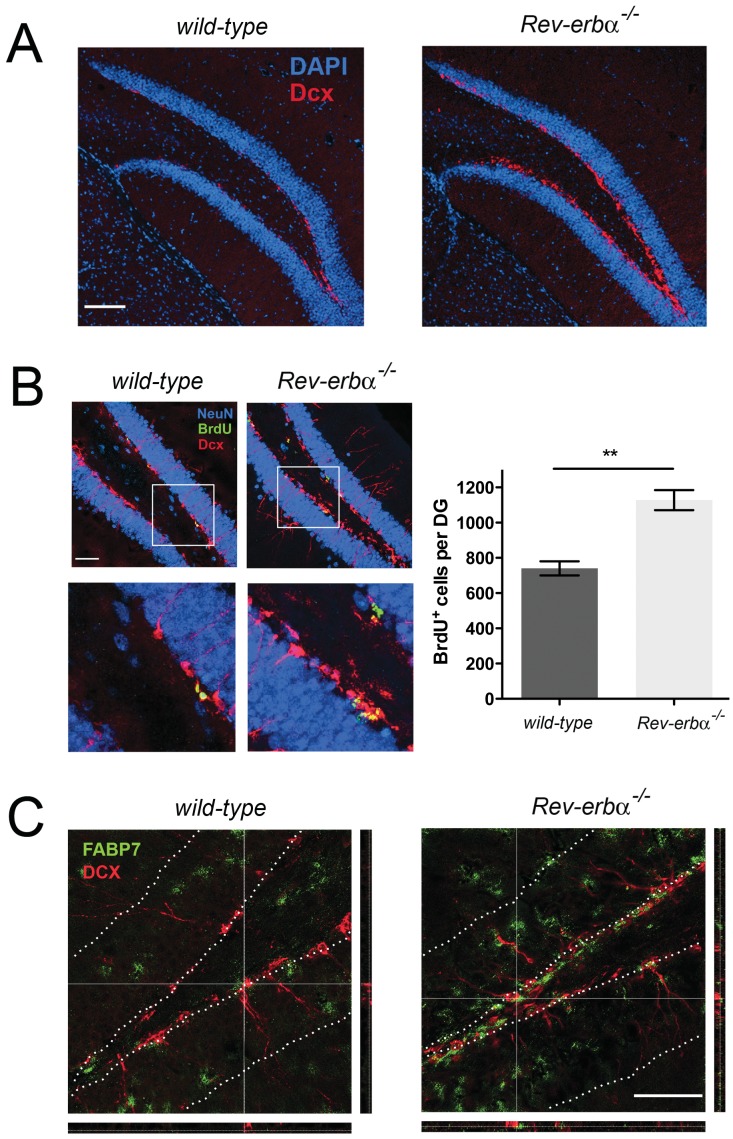
Immunohistochemistry in the dentate gyrus (DG) of wild-type and *Rev-erbα^−/−^* mice at ZT6. (**A**) Cell nuclei stained with DAPI are in blue and antibodies recognizing doublecortin (Dcx) are in red. Dcx expression is increased in the subgranular zone of *Rev-erbα^−/−^* mice indicating the presence of more neuroblasts in these animals. Scale bar: 100 µm. (**B**) Left panel: Visualization of cell division using bromodeoxyuridine (BrdU). Antibodies recognizing NeuN in blue mark nuclei of mature neurons, antibodies recognizing Dcx are in red and antibodies against BrdU are in green. Scale bar: 50 µm. Right panel: Quantification of the BrdU^+^ cells after 4 days. *Rev-erbα^−/−^* mice display more BrdU positive cells (mean ± SEM, n = 3, **p<0.005, t-test). (**C**) FABP7 protein expression (green) does not overlap with Dcx protein expression and both expression levels are higher in *Rev-erbα^−/−^* mice. The orthogonal sectioning to the right and at the bottom show reconstructions from a confocal z-stack in xz and yz direction, respectively. The dotted white lines mark the granular layer of the DG. Scale bar: 50 µm.

To test this hypothesis we performed immunohistochemistry to visualize the formation of neurons in the SGZ of the hippocampal DG. We found that expression of doublecortin (Dcx), a marker for immature neurons, is increased in the *Rev-erbα* KO hippocampus (Fig. 5A). Furthermore, an increased number of cells has divided in *Rev-erbα* KO animals, as evidenced by the cell-cycle dependent incorporation of bromodeoxyuridine (BrdU) (Fig. 5B). Dcx staining and BrdU staining partially overlapped (Fig. 5B, magnification), consistent with the accepted model of neurogenesis [32]. In the molecular layer of the hippocampus a partial overlap in expression was observed between FABP7 and GFAP (Fig. S3). This is consistent with previous observations describing FABP7 as a marker for a subpopulation of glial cells [54].

Since we observed increased *Fabp7* mRNA expression in the SGZ of *Rev-erbα* KO mice (Fig. 2B) we investigated its expression at the protein level. Similar to its mRNA expression, more FABP7 protein containing cells were observed in the SGZ of *Rev-erbα* KO mice (Fig. 5C). Its expression did not co-localize with Dcx, indicating that FABP7 may be a marker of a subpopulation of neuronal stem cells (NSCs, Type-2 and Type-3) and early transitory amplifying cells (TAPs) before Dcx starts to be expressed in neuroblasts [32]. Overall these results suggest a correlation between neurogenesis, FABP7 and REV-ERBα function.

### The diurnal pattern of hippocampal neurogenesis is lost and constantly high in *Rev-erbα* KO mice

**Figure 6:**
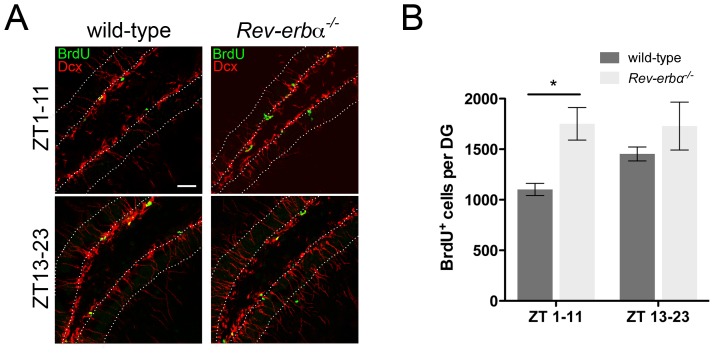
Neurogenesis in *Rev-erbα^−/−^* mice is constantly high and not diurnal. (**A**) BrdU was injected at ZT1 (upper panels) or ZT13 (lower panels) and incorporation was assessed 10 hours later (ZT11 and ZT23, respectively) in wild-type (left panels) and *Rev-erbα^−/−^* KO (right panels). Antibodies recognizing Dcx are in red and antibodies against BrdU are in green. The dotted white lines mark the granular layer of the DG. Scale bar: 50 µm. (**B**) Quantification of the BrdU^+^ cells after 10 hours. *Rev-erbα^−/−^* mice display more BrdU positive cells during the light phase (ZT1-11) compared to wild-type (mean ± SEM, n = 4, *p<0.05, 2-way ANOVA) whereas in the dark phase (ZT13-23) no difference in the number of BrdU positive cells was observed between the genotypes.

Because REV-ERBα appears to be responsible for the diurnal expression of the NPC marker *Fabp7* (Fig. 2, 3), we tested whether the known diurnal pattern of neurogenesis [23]–[26] is lost in FABP7 overexpressing *Rev-erbα* KO mice. In wild-type animals we observed a time of day dependent BrdU incorporation into newborn cells in the SGZ of the hippocampus with higher incorporation during the dark phase (ZT13-23) as compared to the light phase (ZT1-11) (Fig. 6A left panel, 6B). In contrast incorporation of BrdU into newly formed cells of *Rev-erbα* KO was constantly high and did not show a diurnal pattern. This observation correlates with the observed constant overexpression of *Fabp7* in *Rev-erbα* KO animals, suggesting that *Rev-erbα* is involved in establishing the diurnal pattern of adult neurogenesis.

### Migration and proliferation of glioblastoma cells are modulated by *Rev-erbα* and *Fabp7* in vitro

Approximately 80% of dividing progenitors in the SGZ are directed to the neuronal fate and develop into dentate granule neurons. They migrate radially into the inner third of the granule layer where they start to display the morphology of mature granule neurons (reviewed in [55]). Hence, migration is part of adult neurogenesis. In order to establish a functional link between increased expression of FABP7 in *Rev-erbα* KO mice and migration of neuronal cells, we looked at the migration properties of FABP7 expressing U-251 MG glioblastoma cells [36] in a transwell migration assay.

**Figure 7:**
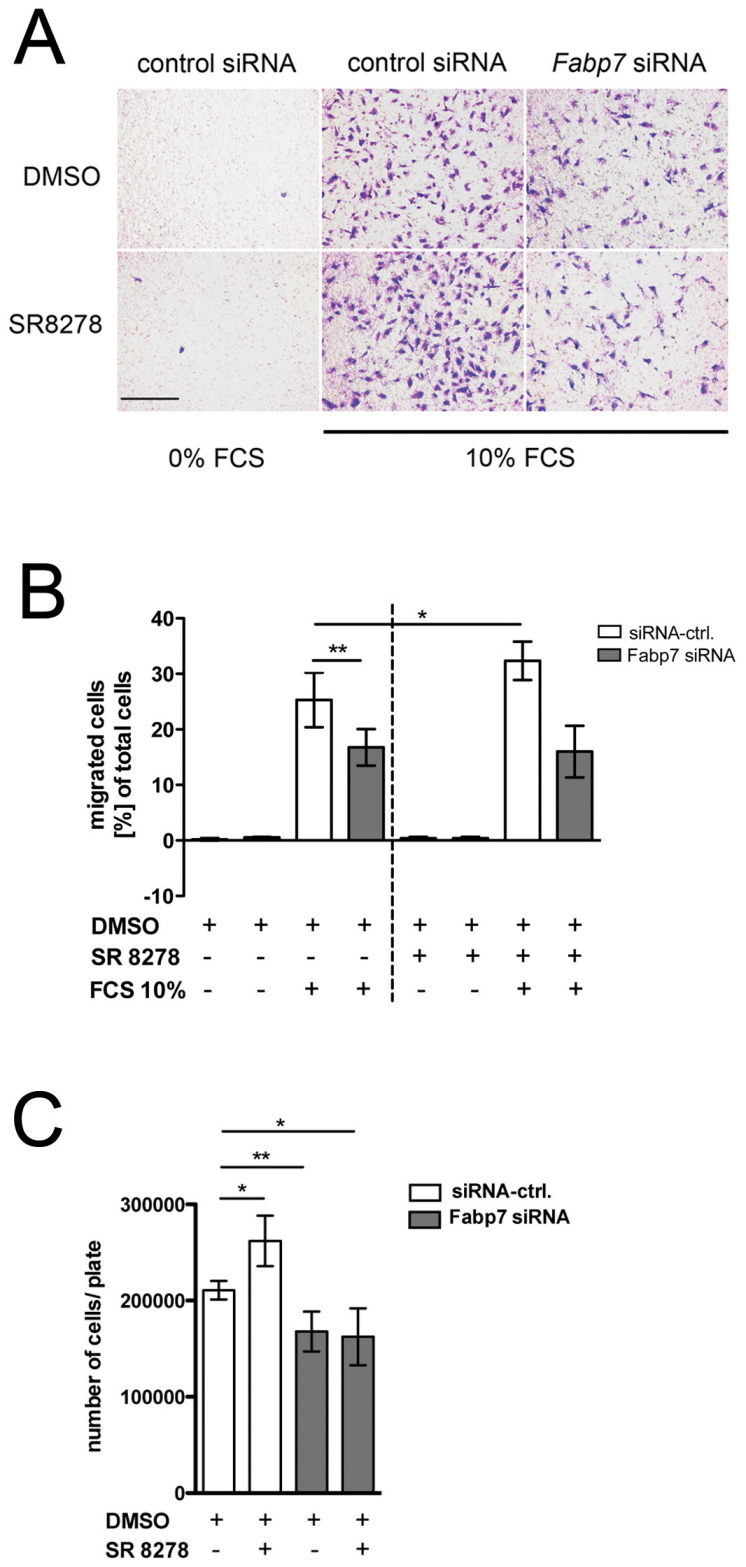
Influence of *Rev-erbα* and *Fabp7* on migration and proliferation of U-251 MG glioblastoma cells. (**A**) Cells were analyzed under non-migrating (0% FCS) and migrating (10% FCS) conditions 6 hours after treatment. The *Rev-erbα* antagonist SR8278 (10 µM) increased migration of the cells compared to DMSO control. siRNA against *Fabp7* (Fabp7 16) reduced this migration. Scale bar: 200 µm. (**B**) Quantification of the experiment in A. Shown is the mean ± SD for n = 3 independent experiments (*p<0.05, **p<0.005, t-test). (**C**) Number of cells 72 hours after transfection with either control or Fabp7 siRNA in presence or absence of the *Rev-erbα* antagonist SR 8278. The mean ± SD for n = 4 experiments (*p<0.05, **p<0.01, t-test) is shown.

Migration of the cells through micropores from one compartment to the other was observed if the latter contained 10% fetal calf serum (FCS). In contrast, no migration was observed if it was left serum-free (Fig. 7A, B). Addition of the REV-ERBα antagonist SR8278 [56] increased FABP7 expression (Fig. S3A) and migration of the cells compared to the solvent control DMSO, indicating that suppression of REV-ERBα had a positive influence on the migration properties of U-251 MG glioblastoma cells (Fig. 7A, B). Introduction of siRNA against *Fabp7* into the cells suppressed both FABP7 expression (Fig. S3B) and migration in the absence and presence of SR8278, (Fig. 7A, B) further supporting the notion that *Rev-erbα* modulates migration via *Fabp7*.

Another hallmark of neurogenesis is cell proliferation. Therefore we tested in the same glioblastoma cell line whether the REV-ERBα antagonist SR8278 and siRNA against *Fabp7* can affect proliferation. 72 hours after transfection of control and *Fabp7* siRNAs, respectively, we counted the number of cells that have grown in presence or absence of SR8278. Suppression of REV-ERBα by its antagonist SR8278 increased proliferation, whereas down regulation of FABP7 by its siRNA decreased it (Fig. 7C). These results were in agreement with our in vivo finding that lack of *Rev-erbα* in mice increased proliferation in the DG (Fig. 5B). This indicates that our observations in U-251 MG glioblastoma cells are likely to be applicable to the DG. Overall it appears that *Rev-erbα* and *Fabp7* are involved in the regulation of proliferation and migration during the process of neurogenesis.

## Discussion

In this study we used genome wide profiling to compare gene expression in the SCN of wild-type and *Rev-erbα* KO mice. We identified *Fabp7* as a direct target gene of REV-ERBα. In situ hybridization and immunohistochemistry revealed an increase in FABP7 expression in *Rev-erbα* KO mice in several brain regions including the SGZ of the hippocampus, suggesting an involvement of REV-ERBα and FABP7 in adult neurogenesis. In accordance with this notion *Rev-erbα* KO mice displayed constantly high proliferation of cells over the day compared to wild-type mice, which displayed a diurnal pattern of neurogenesis in the SGZ. In addition, in vitro manipulation of REV-ERBα and FABP7 affected migration and proliferation properties of glioblastoma cells.

Gene expression profiling of NSCs and their neuronal progeny in adult hippocampal tissue revealed many genes to be involved in neurogenesis [7]. Interestingly, this study indicated that in NSCs *Rev-erbα* (*Nr1d1*) is about 4 times up-regulated compared to immature neurons expressing Dcx [7], suggesting an involvement of *Rev-erbα* in the early steps of adult neurogenesis. Among the potential target genes of *Rev-erbα* identified in our study (Fig. 1, Table S1), *Fabp7* was found to affect neuronal differentiation [57]. However, because *Fabp7* levels are low in NSCs (type 1) and absent in Dcx expressing immature neurons (Fig. 5C, [32]) the study by Bracko et al. [7] did not identify *Fabp7* to be differentially expressed between NSCs and immature neurons.

Analysis of *Fabp7* over 24 hours revealed a diurnal expression of its mRNA in brain tissue which is comparable to a previous study [33] with a trough of expression around ZT16 that is almost anti-phasic to the expression of *Rev-erbα* (Fig. 2C). Although FABP7 protein levels do not fluctuate over time, lack of *Rev-erbα* significantly increased FABP7 protein levels in the brain (Fig. 2D). Our transactivation and ChIP studies indicate that REV-ERBα is a regulator of *Fabp7* mRNA expression (Fig. 3). Thus FABP7 appears to be one of the mediators of REV-ERBα function in the brain.

Lack of *Fabp7* in mice leads to altered emotional behavioral responses [54] and has been associated with a schizophrenia endophenotype [46]. In particular, *Fabp7* KO mice exhibited a differential response in the PPI test accompanied by reduced proliferation in the SGZ [46]. In *Rev-erbα* KO mice, which overexpress *Fabp7* (Fig. 2), the opposite phenotype with increased proliferation in the SGZ (Fig. 5B, 6) and no change in the PPI test (Fig. 4A) was observed. Similarly, anxiety related behavior is altered in *Fabp7* KO mice [54] but in the *Fabp7* overexpressing *Rev-erbα* KO mice this is not the case (Fig. 4B). Furthermore, *Rev-erbα* KO animals did not differ in the tail suspension test (TST) compared to wild-type, however, they responded differently to the FST, spending less time immobile than their wild-type counterparts (Fig. 4D). Decreased immobility has been associated with mania while increased immobility with depression [48] and reduced neurogenesis [53]. Accordingly, one would expect an increase of neurogenesis in *Rev-erbα* KO mice. Our results support this notion as more Dcx positive neuroblasts are observed in the SGZ of *Rev-erbα* KO animals (Fig. 5A). However, the effect of REV-ERBα regulation via FABP7 appears to manifest before neuroblasts are committed, as FABP7 expression did not co-localize with Dcx positive cells (Fig. 5C). This is consistent with previous observations describing a transitory expression of FABP7 in type 2 and 3 NSCs and early TAPs but not in late TAPs and Dcx positive neuroblasts in the SVZ [32].

Adult neurogenesis in the hippocampus has also been associated with learning and memory (for review see [55], [58]). The short-term memory and long-term memory tests we applied to the *Rev-erbα* KO mice revealed, that these animals had a deficit in the process of memory formation (Fig. 4E, F). An increase in adult neurogenesis in the hippocampus, as observed in *Rev-erbα* KO animals would, however, predict an improvement of memory formation. This contradiction may be rooted in the multiple functions of *Rev-erbα*. Our microarray analysis (Fig. 1, Tables S1 and S2) clearly shows that many genes are altered in their expression in *Rev-erbα* KO mice. Of special interest in this context is the down-regulation of the nuclear glucocorticoid receptor (*Nr3c1*), because decreased signaling of this receptor in the hippocampus impaired spatial memory in rats [59], [60]. Interestingly, a recent study shows that adult hippocampal neurogenesis regulates forgetting indicating that too much neurogenesis may jeopardize memory retention [61]. This notion correlates with our findings.

The challenge for NSCs as for any other type of stem cells is to keep the balance between proliferation and quiescence. This balance is extremely important not only to keep a certain amount of pluripotent NSCs in their niche, but also to avoid cancer development due to over-proliferation. Niche signals, such as notch signaling, can control dormant NSCs and push them towards proliferative or keep them in a quiescent state [62]. *Fabp7* may serve as a potential marker for mitotically activated NSCs in the SVZ [32]. *Rev-erbα*, as a repressor of *Fabp7*, may provide an additional niche stimulus and therefore function as a brake to avoid excessive proliferation of NSCs. This may explain why we observe a strong increase in neurogenesis in the SGZ of *Rev-erbα* KO animals. If proliferation is constantly increased, gliomas may develop, which are the most common primary malignancy in the central nervous system of humans. In mice, however, gliomas are hardly observed and we did not note development of gliomas in *Rev-erbα* KO mice. This may be due to compensation of increased neurogenesis by elevation of cell death and/or apoptosis in *Rev-erbα* KO animal, which has been observed in the developing cerebellum of these mice [63]. Alterations in cell death and apoptosis in the adult hippocampus will have to be investigated in *Rev-erbα* KO mice in the future.

Glioblastoma tumors appear to contain cells with stem cell-like properties, which contribute to invasion and chemoresistance [64], [65]. The stem cell-like cells grow as neurospheres in culture and in comparison to adherent glioblastoma cells, they express elevated levels of *Fabp7* accompanied by elevated migration and proliferation [57]. Manipulation of *Rev-erbα* and *Fabp7* in U-251 MG glioblastoma cells shows effects on migration and proliferation (Fig. 7) that are in line with the observations described above. Hence, it can be speculated that agonists for REV-ERBαmay help to reduce proliferation and migration in gliomas, which may represent a novel avenue to combat this type of tumors in humans. Interestingly, it appears that circadian genes are to some extent related to glioma risk and outcome [66]. In particular elevated levels of CLOCK contribute to cell proliferation and migration in glioma [67]. Of note is that *Clock* is directly regulated by REV-ERBα [45] and therefore agonists for REV-ERBα [4] may not only reduce *Fabp7* expression but also *Clock* levels, which may reduce neurogenesis and lower the potential of glioblastoma development.

Neurogenesis in the brain continuously declines with age [68]. This might be partially due to an increased quiescence of NSCs and loss of *Fabp7* expressing cells, as it has been observed in the SVZ of aged mice [32]. Increasing *Fabp7* by the application of REV-ERBα antagonists may awake potentially dormant NSCs in neurogenic pools and they may replenish dying cells. Hence, REV-ERBα antagonists may improve the performance of the ageing brain and help in the treatment of neurodegenerative diseases such as Alzheimer disease [58].

Furthermore, antagonists for REV-ERBα may serve as anti-depressants by increasing proliferation and migration (Fig. 7) leading to a reduction of depressive symptoms (Fig. 4D). However, future experiments will show to what extent the above predictions can be met.

Overall our study shows that *Rev-erbα* regulates *Fabp7* and that both genes appear to be involved in the modulation of neurogenesis. This finding has far reaching implications, as pharmacological targeting of *Rev-erbα* may lead to improved treatments for gliomas as well as neurological and depressive disorders.

## Supporting Information

Figure S1 Expression profile of *Fabp7* mRNA and protein in brain tissue. (**A**) Dark-field microscopy of the SCN and the habenula (HB) comparing wild-type and *Rev-erbα^−/−^* mice at ZT4 and ZT16. The yellow signal represents the hybridization signal detecting *Fabp7* mRNA and blue represents Hoechst-dye stained cell nuclei. (**B**) Quantification of the signal in the SCN, the HB and cortex (CX) over time: black line  =  wild-type, red line  =  *Per2^Brdm1^*, green line  =  *Rev-erbα^−/−^*. The signal at ZT4 is double plotted. The values comparing wild-type (or *Per2^Brdm1^*) with *Rev-erbα^−/−^* are significantly different (n = 3, p<0.05, 2-way ANOVA, mean ± SEM).
(TIF)

Figure S2 Inhibition of *Fabp7* transcription by REV-ERBα in NIH 3T3 fibroblasts. Transactivation experiments show a dose dependent repression potential of REV-ERBα that is similar for both the *Bmal1::luc* and *Fabp7::luc* reporter constructs (n = 3, *p<0.05, mean ± SD).
(TIF)

Figure S3 Immunohistochemistry in the dentate gyrus (DG) of wild-type and *Rev-erbα* ^−/−^ mice at ZT6. Overlapping signals (yellow) of FABP7 (green) with GFAP expressing cells (red). The orthogonal sectioning to the right and on the bottom depict reconstructions from a confocal z-stack in xz and yz direction to confirm that the FABP7 signal cell belongs in fact to the GFAP-positive cell. Scale bar: 50 µm.
(TIF)

Figure S4 Immunobots showing efficiency of inhibition of REV-ERBα activity and verification of siRNA knockdown of *Fabp7* in U-251 MG glioblastoma cells. (**A**) Quantification of FABP7 and BMAL1 protein expression after treatment for 24 h with different concentrations of REV-ERBα antagonist SR8278. Actin was used for normalization. (n = 3, *p<0.05, mean ± SD, t-test). (**B**) Left panel: Comparison of *Fabp7* knock down efficiency between three different siRNAs against *Fabp7* (16, 17, 18) and negative control siRNA. Efficiency was tested 72 h post transfection with siRNA and actin was used for normalization control. The fold change of FABP7 protein expression was calculated setting the control siRNA to 1 (n = 4, **p<0.01, mean ± SD, t-test). Right panel: Quantification of *Fabp7* expression 72 h after siRNA knock down or 18 h after treatment with 10 µM REV-ERBα antagonist SR8278 by qRT-PCR. The fold change of *Fabp7* mRNA expression was calculated setting the control siRNA or solvent control, DMSO, to 1. Experimental conditions were the same as used for migration and proliferation assays (n = 3, *p<0.05, **p<0.01, ***p<0.001, mean ± SD, t-test).
(TIF)

Table S1 List of genes up-regulated in *Rev-erbα* KO SCN.
(PDF)

Table S2 List of genes down-regulated in *Rev-erbα* KO SCN.
(PDF)

Table S3 List of oligonucleotides used in this study.
(PDF)

## References

[1] Buhr ED, Takahashi JS (2013) Molecular components of the Mammalian circadian clock. Handb Exp Pharmacol 217: 3–27. 10.1007/978-3-642-25950-0_1 PMC3762864 23604473

[2] Bugge A, Feng D, Everett LJ, Briggs ER, Mullican SE, et al (2012) Rev-erbalpha and Rev-erbbeta coordinately protect the circadian clock and normal metabolic function. Genes Dev 26: 657–667. 22474260 10.1101/gad.186858.112 PMC3323877

[3] Cho H, Zhao X, Hatori M, Yu RT, Barish GD, et al (2012) Regulation of circadian behaviour and metabolism by REV-ERB-alpha and REV-ERB-beta. Nature 485: 123–127. 22460952 10.1038/nature11048 PMC3367514

[4] Solt LA, Wang Y, Banerjee S, Hughes T, Kojetin DJ, et al (2012) Regulation of circadian behaviour and metabolism by synthetic REV-ERB agonists. Nature 485: 62–68. 22460951 10.1038/nature11030 PMC3343186

[5] Borgs L, Beukelaers P, Vandenbosch R, Nguyen L, Moonen G, et al (2009) Period 2 regulates neural stem/progenitor cell proliferation in the adult hippocampus. BMC Neurosci 10: 30. 19327139 10.1186/1471-2202-10-30 PMC2714160

[6] Kimiwada T, Sakurai M, Ohashi H, Aoki S, Tominaga T, et al (2009) Clock genes regulate neurogenic transcription factors, including NeuroD1, and the neuronal differentiation of adult neural stem/progenitor cells. Neurochem Int 54: 277–285. 19121353 10.1016/j.neuint.2008.12.005

[7] Bracko O, Singer T, Aigner S, Knobloch M, Winner B, et al (2012) Gene expression profiling of neural stem cells and their neuronal progeny reveals IGF2 as a regulator of adult hippocampal neurogenesis. J Neurosci 32: 3376–3387. 22399759 10.1523/JNEUROSCI.4248-11.2012 PMC3338187

[8] Janich P, Pascual G, Merlos-Suarez A, Batlle E, Ripperger J, et al (2011) The circadian molecular clock creates epidermal stem cell heterogeneity. Nature 480: 209–214. 22080954 10.1038/nature10649

[9] Janich P, Toufighi K, Solanas G, Luis NM, Minkwitz S, et al (2013) Human epidermal stem cell function is regulated by circadian oscillations. Cell Stem Cell 13: 745–753. 24120744 10.1016/j.stem.2013.09.004

[10] Lie DC, Song H, Colamarino SA, Ming GL, Gage FH (2004) Neurogenesis in the adult brain: new strategies for central nervous system diseases. Annu Rev Pharmacol Toxicol 44: 399–421. 14744252 10.1146/annurev.pharmtox.44.101802.121631

[11] Zhao C, Deng W, Gage FH (2008) Mechanisms and functional implications of adult neurogenesis. Cell 132: 645–660. 18295581 10.1016/j.cell.2008.01.033

[12] Samuels BA, Hen R (2011) Neurogenesis and affective disorders. Eur J Neurosci 33: 1152–1159. 21395859 10.1111/j.1460-9568.2011.07614.x

[13] Lois C, Alvarez-Buylla A (1994) Long-distance neuronal migration in the adult mammalian brain. Science 264: 1145–1148. 8178174 10.1126/science.8178174

[14] Kuhn HG, Dickinson-Anson H, Gage FH (1996) Neurogenesis in the dentate gyrus of the adult rat: age-related decrease of neuronal progenitor proliferation. J Neurosci 16: 2027–2033. 8604047 10.1523/JNEUROSCI.16-06-02027.1996 PMC6578509

[15] Gould E, McEwen BS, Tanapat P, Galea LA, Fuchs E (1997) Neurogenesis in the dentate gyrus of the adult tree shrew is regulated by psychosocial stress and NMDA receptor activation. J Neurosci 17: 2492–2498. 9065509 10.1523/JNEUROSCI.17-07-02492.1997 PMC6573503

[16] Gould E, Tanapat P, Cameron HA (1997) Adrenal steroids suppress granule cell death in the developing dentate gyrus through an NMDA receptor-dependent mechanism. Brain Res Dev Brain Res 103: 91–93. 9370064 10.1016/s0165-3806(97)00079-5

[17] van Praag H, Kempermann G, Gage FH (1999) Running increases cell proliferation and neurogenesis in the adult mouse dentate gyrus. Nat Neurosci 2: 266–270. 10195220 10.1038/6368

[18] Guzman-Marin R, Suntsova N, Stewart DR, Gong H, Szymusiak R, et al (2003) Sleep deprivation reduces proliferation of cells in the dentate gyrus of the hippocampus in rats. J Physiol 549: 563–571. 12679377 10.1113/jphysiol.2003.041665 PMC2342950

[19] Kempermann G, Kuhn HG, Gage FH (1997) More hippocampal neurons in adult mice living in an enriched environment. Nature 386: 493–495. 9087407 10.1038/386493a0

[20] Gibson EM, Wang C, Tjho S, Khattar N, Kriegsfeld LJ (2010) Experimental ‘jet lag’ inhibits adult neurogenesis and produces long-term cognitive deficits in female hamsters. PLoS One 5: e15267. 21152025 10.1371/journal.pone.0015267 PMC2995744

[21] Kott J, Leach G, Yan L (2012) Direction-dependent effects of chronic “jet-lag” on hippocampal neurogenesis. Neurosci Lett 515: 177–180. 22465247 10.1016/j.neulet.2012.03.048

[22] Golombek DA, Rosenstein RE (2010) Physiology of circadian entrainment. Physiol Rev 90: 1063–1102. 20664079 10.1152/physrev.00009.2009

[23] Goergen EM, Bagay LA, Rehm K, Benton JL, Beltz BS (2002) Circadian control of neurogenesis. J Neurobiol 53: 90–95. 12360586 10.1002/neu.10095

[24] Kochman LJ, Weber ET, Fornal CA, Jacobs BL (2006) Circadian variation in mouse hippocampal cell proliferation. Neurosci Lett 406: 256–259. 16930842 10.1016/j.neulet.2006.07.058

[25] Tamai S, Sanada K, Fukada Y (2008) Time-of-day-dependent enhancement of adult neurogenesis in the hippocampus. PLoS One 3: e3835. 19048107 10.1371/journal.pone.0003835 PMC2585014

[26] Gilhooley MJ, Pinnock SB, Herbert J (2011) Rhythmic expression of per1 in the dentate gyrus is suppressed by corticosterone: implications for neurogenesis. Neurosci Lett 489: 177–181. 21163331 10.1016/j.neulet.2010.12.011

[27] Preitner N, Damiola F, Lopez-Molina L, Zakany J, Duboule D, et al (2002) The orphan nuclear receptor REV-ERBalpha controls circadian transcription within the positive limb of the mammalian circadian oscillator. Cell 110: 251–260. 12150932 10.1016/s0092-8674(02)00825-5

[28] Chmurzynska A (2006) The multigene family of fatty acid-binding proteins (FABPs): function, structure and polymorphism. J Appl Genet 47: 39–48. 16424607 10.1007/BF03194597

[29] Feng L, Hatten ME, Heintz N (1994) Brain lipid-binding protein (BLBP): a novel signaling system in the developing mammalian CNS. Neuron 12: 895–908. 8161459 10.1016/0896-6273(94)90341-7

[30] Yun SW, Leong C, Zhai D, Tan YL, Lim L, et al (2012) Neural stem cell specific fluorescent chemical probe binding to FABP7. Proc Natl Acad Sci U S A 109: 10214–10217. 22689954 10.1073/pnas.1200817109 PMC3387064

[31] Young JK, Heinbockel T, Gondre-Lewis MC (2013) Astrocyte fatty acid binding protein-7 is a marker for neurogenic niches in the rat hippocampus. Hippocampus 23: 1476–1483. 23996503 10.1002/hipo.22200 PMC3859315

[32] Giachino C, Basak O, Lugert S, Knuckles P, Obernier K, et al (2014) Molecular diversity subdivides the adult forebrain neural stem cell population. Stem Cells 32: 70–84. 23964022 10.1002/stem.1520 PMC4259462

[33] Gerstner JR, Bremer QZ, Vander Heyden WM, Lavaute TM, Yin JC, et al (2008) Brain fatty acid binding protein (Fabp7) is diurnally regulated in astrocytes and hippocampal granule cell precursors in adult rodent brain. PLoS One 3: e1631. 18286188 10.1371/journal.pone.0001631 PMC2238817

[34] Gerstner JR, Vanderheyden WM, LaVaute T, Westmark CJ, Rouhana L, et al (2012) Time of day regulates subcellular trafficking, tripartite synaptic localization, and polyadenylation of the astrocytic Fabp7 mRNA. J Neurosci 32: 1383–1394. 22279223 10.1523/JNEUROSCI.3228-11.2012 PMC3564590

[35] Hamprecht B (1977) Structural, electrophysiological, biochemical, and pharmacological properties of neuroblastoma-glioma cell hybrids in cell culture. Int Rev Cytol 49: 99–170. 16829 10.1016/s0074-7696(08)61948-8

[36] Ponten J, Westermark B (1978) Properties of human malignant glioma cells in vitro. Med Biol 56: 184–193. 359950

[37] Bolstad BM, Irizarry RA, Astrand M, Speed TP (2003) A comparison of normalization methods for high density oligonucleotide array data based on variance and bias. Bioinformatics 19: 185–193. 12538238 10.1093/bioinformatics/19.2.185

[38] Albrecht U, Sun ZS, Eichele G, Lee CC (1997) A differential response of two putative mammalian circadian regulators, mper1 and mper2, to light. Cell 91: 1055–1064. 9428527 10.1016/s0092-8674(00)80495-x

[39] Schmutz I, Ripperger JA, Baeriswyl-Aebischer S, Albrecht U (2010) The mammalian clock component PERIOD2 coordinates circadian output by interaction with nuclear receptors. Genes Dev 24: 345–357. 20159955 10.1101/gad.564110 PMC2816734

[40] Langmesser S, Tallone T, Bordon A, Rusconi S, Albrecht U (2008) Interaction of circadian clock proteins PER2 and CRY with BMAL1 and CLOCK. BMC Mol Biol 9: 41. 18430226 10.1186/1471-2199-9-41 PMC2383916

[41] Ripperger JA, Schibler U (2006) Rhythmic CLOCK-BMAL1 binding to multiple E-box motifs drives circadian Dbp transcription and chromatin transitions. Nat Genet 38: 369–374. 16474407 10.1038/ng1738

[42] Stratmann M, Stadler F, Tamanini F, van der Horst GT, Ripperger JA (2010) Flexible phase adjustment of circadian albumin D site-binding protein (DBP) gene expression by CRYPTOCHROME1. Genes Dev 24: 1317–1328. 20551177 10.1101/gad.578810 PMC2885666

[43] Braff DL, Geyer MA (1990) Sensorimotor gating and schizophrenia. Human and animal model studies. Arch Gen Psychiatry 47: 181–188. 2405807 10.1001/archpsyc.1990.01810140081011

[44] Reppert SM, Weaver DR (2002) Coordination of circadian timing in mammals. Nature 418: 935–941. 12198538 10.1038/nature00965

[45] Crumbley C, Burris TP (2011) Direct regulation of CLOCK expression by REV-ERB. PLoS One 6: e17290. 21479263 10.1371/journal.pone.0017290 PMC3066191

[46] Watanabe A, Toyota T, Owada Y, Hayashi T, Iwayama Y, et al (2007) Fabp7 maps to a quantitative trait locus for a schizophrenia endophenotype. PLoS Biol 5: e297. 18001149 10.1371/journal.pbio.0050297 PMC2071943

[47] Hampp G, Ripperger JA, Houben T, Schmutz I, Blex C, et al (2008) Regulation of monoamine oxidase A by circadian-clock components implies clock influence on mood. Curr Biol 18: 678–683. 18439826 10.1016/j.cub.2008.04.012

[48] Petit-Demouliere B, Chenu F, Bourin M (2005) Forced swimming test in mice: a review of antidepressant activity. Psychopharmacology (Berl) 177: 245–255. 15609067 10.1007/s00213-004-2048-7

[49] Renard CE, Dailly E, David DJ, Hascoet M, Bourin M (2003) Monoamine metabolism changes following the mouse forced swimming test but not the tail suspension test. Fundam Clin Pharmacol 17: 449–455. 12914547 10.1046/j.1472-8206.2003.00160.x

[50] Lalonde R (2002) The neurobiological basis of spontaneous alternation. Neurosci Biobehav Rev 26: 91–104. 11835987 10.1016/s0149-7634(01)00041-0

[51] Wimmer ME, Hernandez PJ, Blackwell J, Abel T (2012) Aging impairs hippocampus-dependent long-term memory for object location in mice. Neurobiol Aging 33: 2220–2224. 21872364 10.1016/j.neurobiolaging.2011.07.007 PMC3227775

[52] Jager J, O'Brien WT, Manlove J, Krizman EN, Fang B, et al (2014) Behavioral Changes and Dopaminergic Dysregulation in Mice Lacking the Nuclear Receptor Rev-erbalpha. Mol Endocrinol 28: 490–498. 24552589 10.1210/me.2013-1351 PMC3968406

[53] Snyder JS, Soumier A, Brewer M, Pickel J, Cameron HA (2011) Adult hippocampal neurogenesis buffers stress responses and depressive behaviour. Nature 476: 458–461. 21814201 10.1038/nature10287 PMC3162077

[54] Owada Y, Abdelwahab SA, Kitanaka N, Sakagami H, Takano H, et al (2006) Altered emotional behavioral responses in mice lacking brain-type fatty acid-binding protein gene. Eur J Neurosci 24: 175–187. 16882015 10.1111/j.1460-9568.2006.04855.x

[55] Gu Y, Janoschka S, Ge S (2013) Neurogenesis and hippocampal plasticity in adult brain. Curr Top Behav Neurosci 15: 31–48. 22879073 10.1007/7854_2012_217

[56] Kojetin D, Wang Y, Kamenecka TM, Burris TP (2011) Identification of SR8278, a synthetic antagonist of the nuclear heme receptor REV-ERB. ACS Chem Biol 6: 131–134. 21043485 10.1021/cb1002575 PMC3042041

[57] De Rosa A, Pellegatta S, Rossi M, Tunici P, Magnoni L, et al (2012) A radial glia gene marker, fatty acid binding protein 7 (FABP7), is involved in proliferation and invasion of glioblastoma cells. PLoS One 7: e52113. 23284888 10.1371/journal.pone.0052113 PMC3528762

[58] Mu Y, Gage FH (2011) Adult hippocampal neurogenesis and its role in Alzheimer's disease. Mol Neurodegener 6: 85. 22192775 10.1186/1750-1326-6-85 PMC3261815

[59] Bizon JL, Helm KA, Han JS, Chun HJ, Pucilowska J, et al (2001) Hypothalamic-pituitary-adrenal axis function and corticosterone receptor expression in behaviourally characterized young and aged Long-Evans rats. Eur J Neurosci 14: 1739–1751. 11860468 10.1046/j.0953-816x.2001.01781.x

[60] Lee SY, Hwang YK, Yun HS, Han JS (2012) Decreased levels of nuclear glucocorticoid receptor protein in the hippocampus of aged Long-Evans rats with cognitive impairment. Brain Res 1478: 48–54. 22971526 10.1016/j.brainres.2012.08.035

[61] Akers KG, Martinez-Canabal A, Restivo L, Yiu AP, De Cristofaro A, et al (2014) Hippocampal neurogenesis regulates forgetting during adulthood and infancy. Science 344: 598–602. 24812394 10.1126/science.1248903

[62] Basak O, Giachino C, Fiorini E, Macdonald HR, Taylor V (2012) Neurogenic subventricular zone stem/progenitor cells are Notch1-dependent in their active but not quiescent state. J Neurosci 32: 5654–5666. 22514327 10.1523/JNEUROSCI.0455-12.2012 PMC6703480

[63] Chomez P, Neveu I, Mansen A, Kiesler E, Larsson L, et al (2000) Increased cell death and delayed development in the cerebellum of mice lacking the rev-erbA(alpha) orphan receptor. Development 127: 1489–1498. 10704394 10.1242/dev.127.7.1489

[64] Singh SK, Clarke ID, Terasaki M, Bonn VE, Hawkins C, et al (2003) Identification of a cancer stem cell in human brain tumors. Cancer Res 63: 5821–5828. 14522905

[65] Alcantara Llaguno (2009) Chen J, Parada LF (2009) Signaling in malignant astrocytomas: role of neural stem cells and its therapeutic implications. Clin Cancer Res 15: 7124–7129. 19934302 10.1158/1078-0432.CCR-09-0433 PMC2787668

[66] Madden MH, Anic GM, Thompson RC, Nabors LB, Olson JJ, et al (2014) Circadian pathway genes in relation to glioma risk and outcome. Cancer Causes Control 25: 25–32. 24135790 10.1007/s10552-013-0305-y PMC3947318

[67] Li A, Lin X, Tan X, Yin B, Han W, et al (2013) Circadian gene Clock contributes to cell proliferation and migration of glioma and is directly regulated by tumor-suppressive miR-124. FEBS Lett 587: 2455–2460. 23792158 10.1016/j.febslet.2013.06.018

[68] Encinas JM, Michurina TV, Peunova N, Park JH, Tordo J, et al (2011) Division-coupled astrocytic differentiation and age-related depletion of neural stem cells in the adult hippocampus. Cell Stem Cell 8: 566–579. 21549330 10.1016/j.stem.2011.03.010 PMC3286186

