# Supplementary figures and images for: The Nuclear Receptor REV-ERBα Regulates Fabp7 and Modulates Adult Hippocampal Neurogenesis
Source: PLoS One. 2014 Jun 16;9(6):e99883. doi: 10.1371/journal.pone.0099883 (PMC4059695; doi:10.1371/journal.pone.0099883)

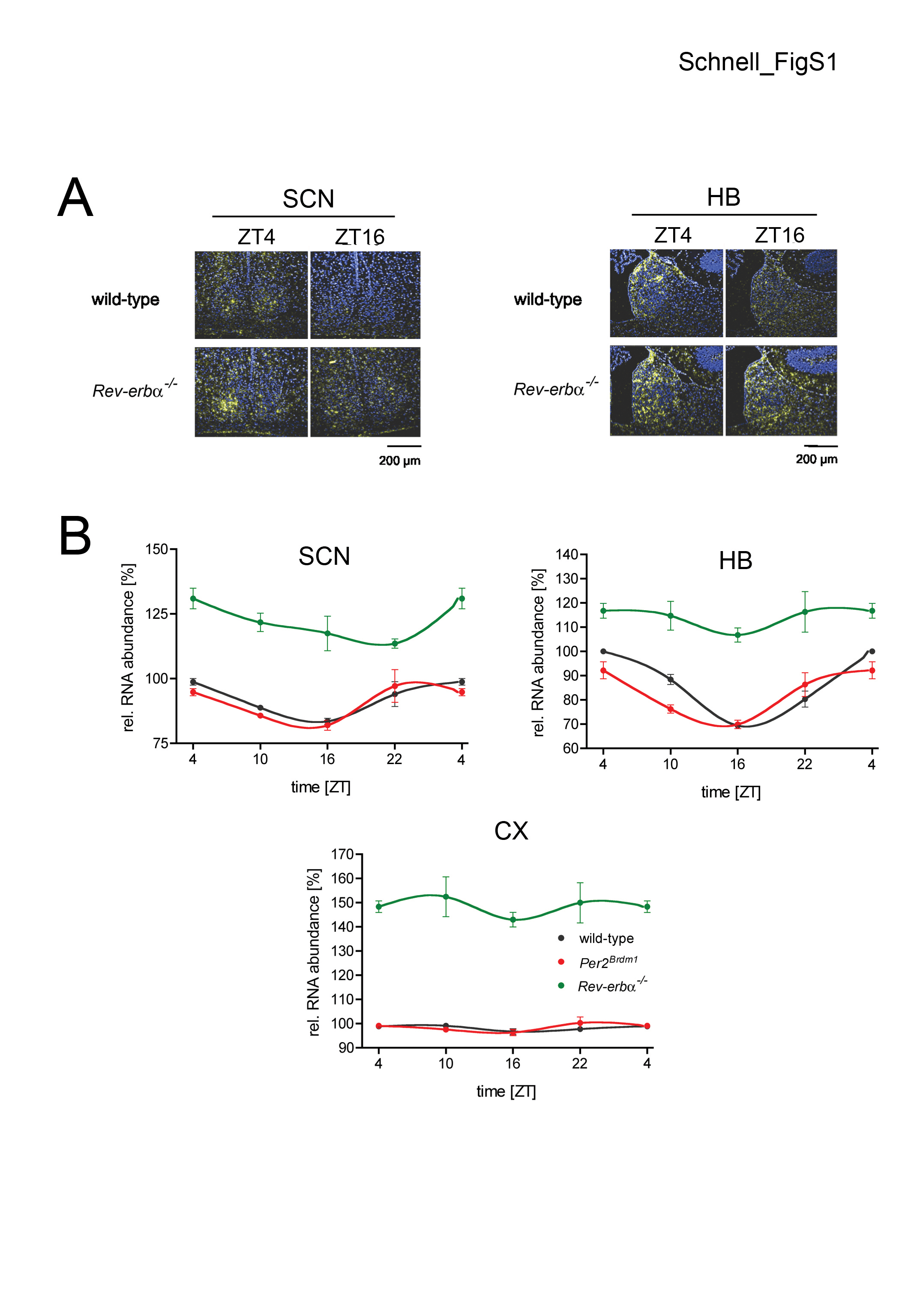

Supplement: Figure S1 — Expression profile of Fabp7 mRNA and protein in brain tissue. (A) Dark-field microscopy of the SCN and the habenula (HB) comparing wild-type and Rev-erbα−/− mice at ZT4 and ZT16. The yellow signal represents the hybridization signal detecting Fabp7 mRNA and blue represents Hoechst-dye stained cell nuclei. (B) Quantification of the signal in the SCN, the HB and cortex (CX) over time: black line = wild-type, red line = Per2Brdm1, green line = Rev-erbα−/−. The signal at ZT4 is double plotted. The values comparing wild-type (or Per2Brdm1) with Rev-erbα−/− are significantly different (n = 3, p<0.05, 2-way ANOVA, mean ± SEM). (TIF) [file pone.0099883.s001.tif]

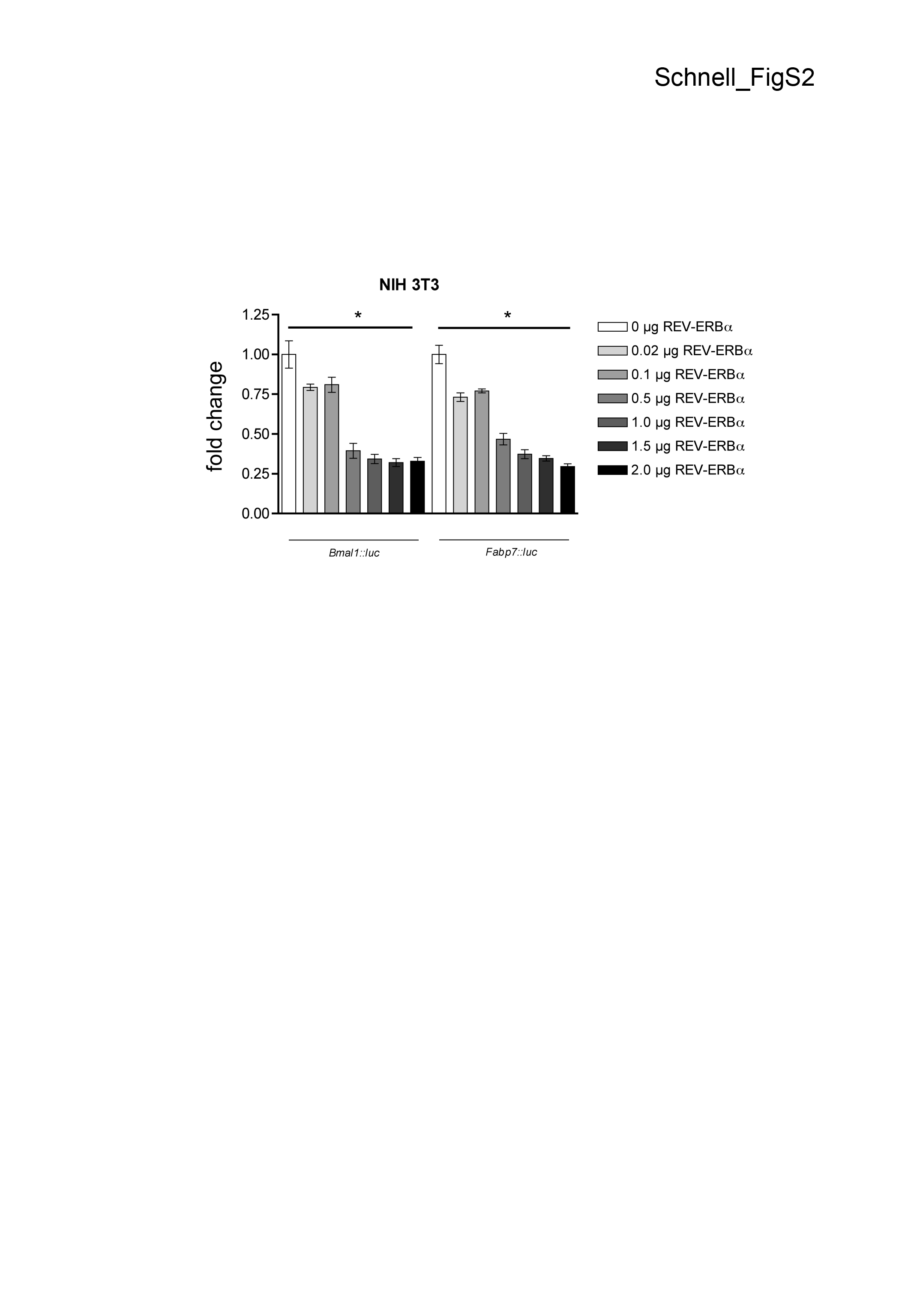

Supplement: Figure S2 — Inhibition of Fabp7 transcription by REV-ERBα in NIH 3T3 fibroblasts. Transactivation experiments show a dose dependent repression potential of REV-ERBα that is similar for both the Bmal1::luc and Fabp7::luc reporter constructs (n = 3, *p<0.05, mean ± SD). (TIF) [file pone.0099883.s002.tif]

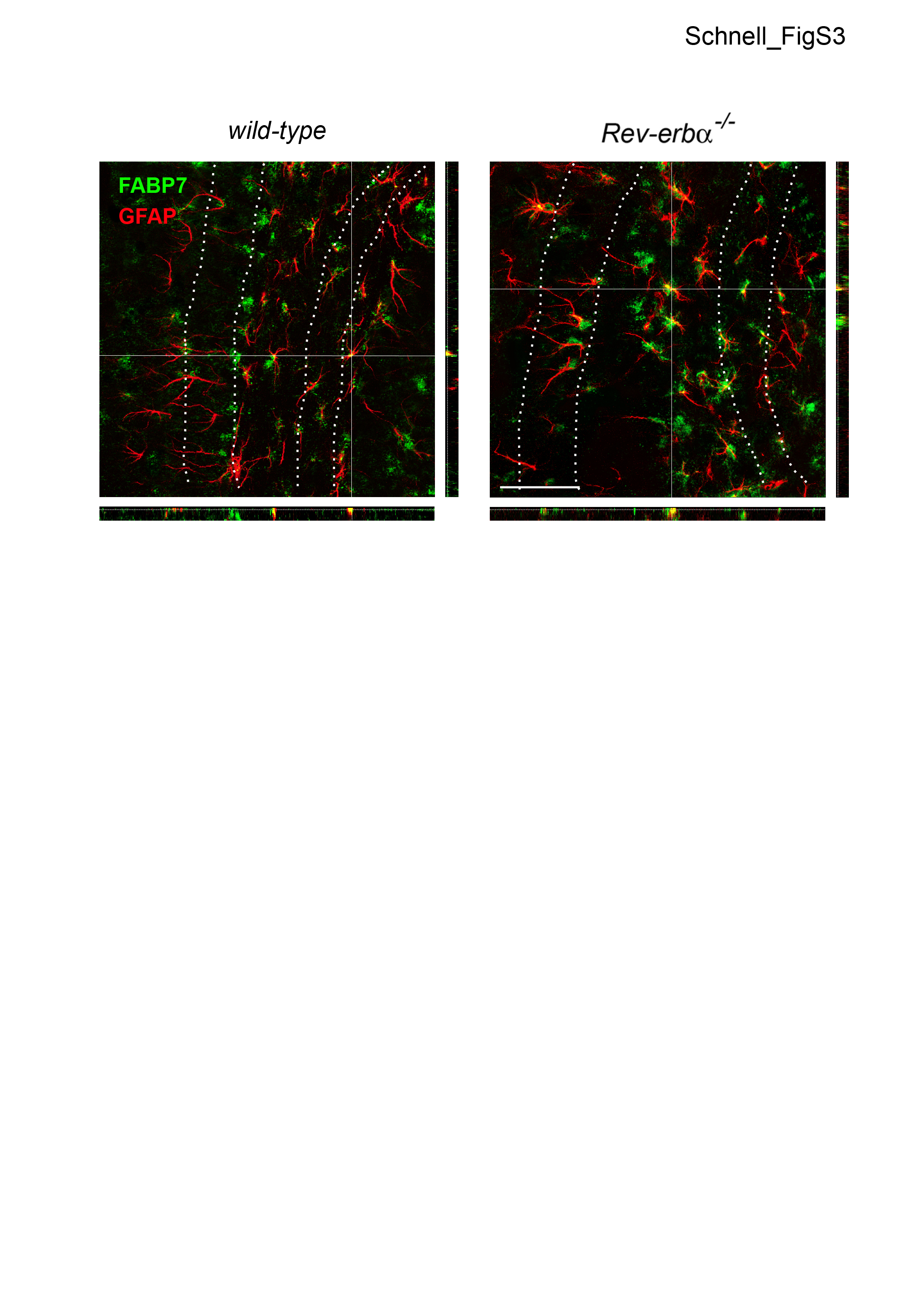

Supplement: Figure S3 — Immunohistochemistry in the dentate gyrus (DG) of wild-type and Rev-erbα −/− mice at ZT6. Overlapping signals (yellow) of FABP7 (green) with GFAP expressing cells (red). The orthogonal sectioning to the right and on the bottom depict reconstructions from a confocal z-stack in xz and yz direction to confirm that the FABP7 signal cell belongs in fact to the GFAP-positive cell. Scale bar: 50 µm. (TIF) [file pone.0099883.s003.tif]

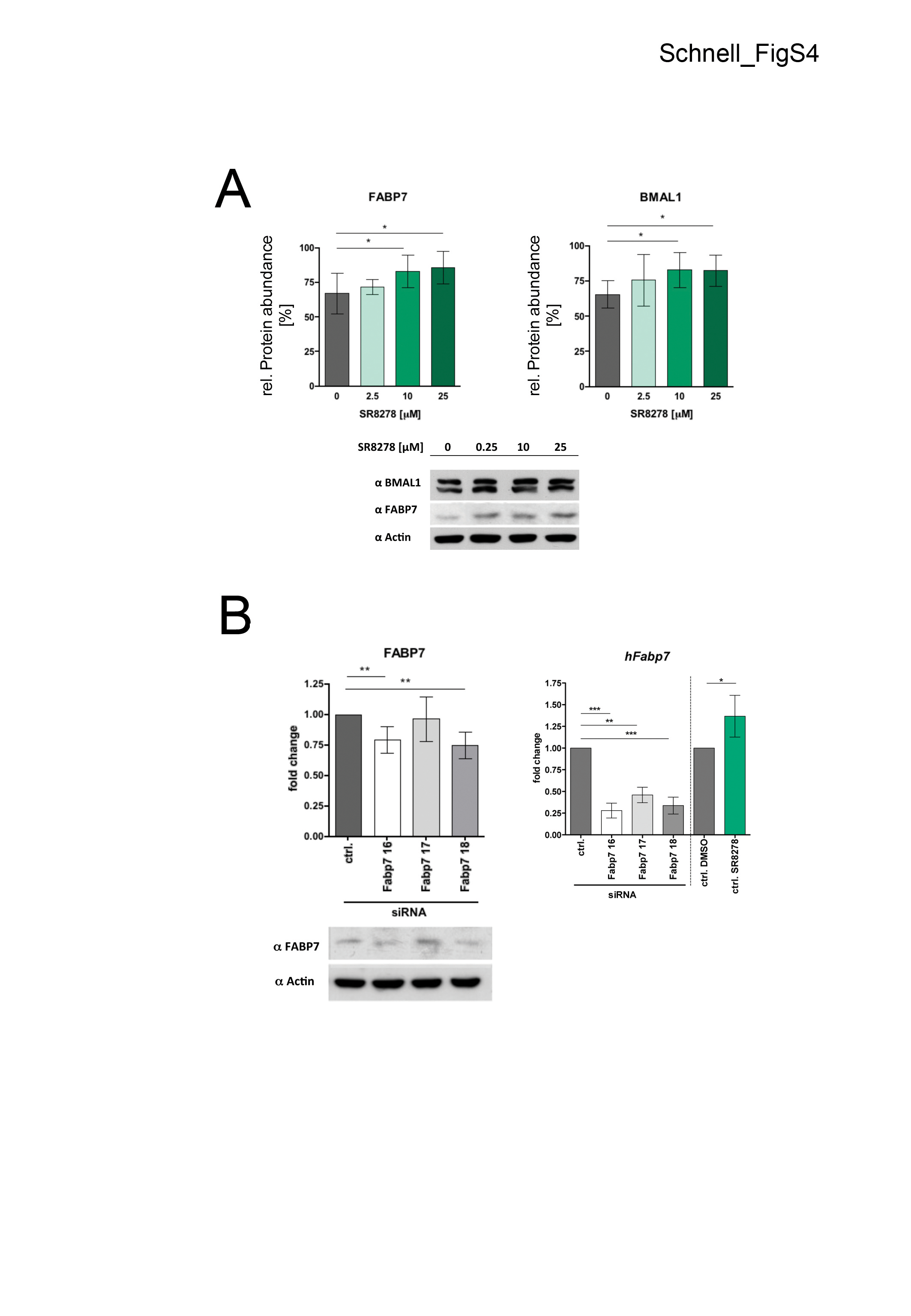

Supplement: Figure S4 — Immunobots showing efficiency of inhibition of REV-ERBα activity and verification of siRNA knockdown of Fabp7 in U-251 MG glioblastoma cells. (A) Quantification of FABP7 and BMAL1 protein expression after treatment for 24 h with different concentrations of REV-ERBα antagonist SR8278. Actin was used for normalization. (n = 3, *p<0.05, mean ± SD, t-test). (B) Left panel: Comparison of Fabp7 knock down efficiency between three different siRNAs against Fabp7 (16, 17, 18) and negative control siRNA. Efficiency was tested 72 h post transfection with siRNA and actin was used for normalization control. The fold change of FABP7 protein expression was calculated setting the control siRNA to 1 (n = 4, **p<0.01, mean ± SD, t-test). Right panel: Quantification of Fabp7 expression 72 h after siRNA knock down or 18 h after treatment with 10 µM REV-ERBα antagonist SR8278 by qRT-PCR. The fold change of Fabp7 mRNA expression was calculated setting the control siRNA or solvent control, DMSO, to 1. Experimental conditions were the same as used for migration and proliferation assays (n = 3, *p<0.05, **p<0.01, ***p<0.001, mean ± SD, t-test). (TIF) [file pone.0099883.s004.tif]
