# Supplementary material for: The Nuclear Receptor REV-ERBα Regulates Fabp7 and Modulates Adult Hippocampal Neurogenesis
Source: PLoS One. 2014 Jun 16;9(6):e99883. doi: 10.1371/journal.pone.0099883 (PMC4059695; doi:10.1371/journal.pone.0099883)
Supplement: Table S1 — List of genes up-regulated in Rev-erbα KO SCN. (PDF) [file pone.0099883.s005.pdf]

Table S1

Probeset ID	Entrez Gene	Gene Symbol	Gene Title	RefSeq Transcript ID	p-value	stepup(p-value)	t	Mean(Rev Erb (--))	Mean(WT)	MeanDiff(Rev Erb (--)-WT)	FoldChange(Rev Erb (--)/WT)	FoldChange(Rev Erb (--)/WT) (Description)
1450779_at	12140	Fabp7	fatty acid bin	NM_021272	1.91E-05	0.172295	37.0984	12.1854	10.0876	2.0978	4.28056	Greater than 0
1415806_at	18791	Plat	plasminogen	NM_008872	0.00014977	0.35551	33.9192	8.74698	8.04574	0.701239	1.6259	Greater than 0
1437056_x_a	78892	Crispld2	cysteine-rich	NM_030209	0.00019083	0.374199	13.3599	8.1312	7.17358	0.957616	1.9421	Greater than 0
1440773_at	382010	BC088983	cDNA sequen	NM_001009	0.00047366	0.417158	10.5234	6.18471	5.51505	0.669655	1.59069	Greater than 0
1421907_at	19014	Med1	mediator cor	NM_001080	0.0005204	0.417158	19.5181	8.82237	7.82005	1.00232	2.00322	Greater than 0
1452975_at	71760	Agxt2l1	alanine-glyo	NM_001163	0.0005284	0.417158	41.1949	10.9419	10.1908	0.7511	1.68308	Greater than 0
1418090_at	84094	Plvap	plasmalemm	NM_032398	0.00072297	0.417158	10.1107	9.39363	8.36452	1.0291	2.04076	Greater than 0
1436115_at	212539	Gm266	predicted ge	NM_001033	0.00143176	0.448151	7.8492	8.07414	6.85337	1.22077	2.33072	Greater than 0
1450725_s_a	23831	Car14	carbonic anh	NM_011797	0.00174933	0.462159	13.101	8.5954	7.48064	1.11476	2.16559	Greater than 0
1460458_at	78892	Crispld2	cysteine-rich	NM_030209	0.00186117	0.463036	17.4074	6.51532	5.85033	0.66499	1.58556	Greater than 0
1441430_at	---	---	---	---	0.00215782	0.474935	8.25034	8.18611	7.39473	0.791379	1.73073	Greater than 0
1421037_at	18143	Npas2	neuronal PAS	NM_008719	0.00267762	0.493072	7.30434	6.33878	5.38711	0.951671	1.93411	Greater than 0
1417130_s_a	57875	Angptl4	angiopoietin-	NM_020581	0.00276587	0.501202	6.68926	7.20628	6.5425	0.663774	1.58422	Greater than 0
1450712_at	16524	Kcnj9	potassium in	NM_008429	0.00277093	0.501202	6.76756	7.0472	5.68973	1.35747	2.56235	Greater than 0
1421679_a_a	12575	Cdkn1a	cyclin-depen	NM_001111	0.00436461	0.511218	8.27631	7.53148	6.88655	0.644929	1.56366	Greater than 0
1436870_s_a	226250	Afap1l2	actin filamen	NM_001177	0.00454795	0.511218	7.01968	7.53911	6.79534	0.743769	1.67454	Greater than 0
1454886_x_a	94090	Trim9	tripartite mo	NM_001110	0.00547535	0.52282	6.59694	9.26429	8.56613	0.698156	1.62243	Greater than 0
1434758_at	78892	Crispld2	cysteine-rich	NM_030209	0.00560114	0.52282	6.46118	6.7046	5.91638	0.788217	1.72694	Greater than 0
1435176_a_a	15902	Id2	inhibitor of D	NM_010496	0.00592162	0.525199	10.2613	10.1823	9.33931	0.842959	1.79373	Greater than 0
1435189_at	666060	Frmpd1	FERM and PD	NM_001081	0.00602684	0.526446	8.64103	7.73445	6.85984	0.874608	1.83351	Greater than 0
1424638_at	12575	Cdkn1a	cyclin-depen	NM_001111	0.00622519	0.533769	6.22729	8.68739	7.75419	0.933201	1.90951	Greater than 0
1457373_at	---	---	---	---	0.00631852	0.535608	5.27497	7.47467	6.77008	0.704592	1.62968	Greater than 0
1423110_at	12843	Col1a2	collagen, typ	NM_007743	0.00754798	0.55235	9.18846	5.86131	5.239	0.622311	1.53934	Greater than 0
1425099_a_a	11865	Arntl	aryl hydrocar	NM_001243	0.00766915	0.55235	10.1276	8.43153	7.67722	0.754313	1.68683	Greater than 0
1447307_at	---	---	---	---	0.0080578	0.558231	5.09358	4.87734	4.13313	0.744215	1.67506	Greater than 0
1448383_at	17387	Mmp14	matrix metal	NM_008608	0.0115719	0.586241	8.93396	8.54713	7.49856	1.04857	2.06848	Greater than 0
1450371_at	22094	Tshb	thyroid stimu	NM_001165	0.0118457	0.586241	6.7474	7.82556	5.84379	1.98177	3.94977	Greater than 0
1433670_at	13731	Emp2	epithelial me	NM_007929	0.0120236	0.586241	5.23603	8.28111	7.46516	0.815946	1.76045	Greater than 0
1435998_at	239083	Ccnb1ip1	cyclin B1 inte	NM_001111	0.0144965	0.601418	6.07221	6.26297	4.56532	1.69765	3.24373	Greater than 0
1422537_a_a	15902	Id2	inhibitor of D	NM_010496	0.0163346	0.618833	5.10731	9.86748	9.21214	0.655346	1.57499	Greater than 0
1456231_at	237625	Pla2g3	phospholipas	NM_172791	0.0167002	0.618833	4.7171	5.43104	4.79071	0.64033	1.55869	Greater than 0
1416572_at	17387	Mmp14	matrix metal	NM_008608	0.017676	0.622453	4.76771	6.62833	5.85898	0.76935	1.7045	Greater than 0

1450784_at	53614	Reck	reversion-ind	NM_016678	0.0177013	0.622453	3.94779	6.78119	6.17917	0.60202	1.51784	Greater than 0
1451901_at	20585	Hltf	helicase-like	NM_009210	0.018316	0.627674	4.03194	4.77893	4.09076	0.688168	1.61124	Greater than 0
1437671_x_a	76453	Prss23	protease, ser	NM_029614	0.0209231	0.634794	3.70598	6.28019	5.61357	0.666627	1.58736	Greater than 0
1442424_at	---	---	---	---	0.0222042	0.646038	3.94146	6.0272	5.28157	0.745628	1.6767	Greater than 0
1458282_at	---	---	---	---	0.0253181	0.653128	3.6794	6.8863	6.2662	0.620109	1.53699	Greater than 0
1460604_at	73649	Cybrd1	cytochrome	NM_028593	0.0284839	0.668046	3.59038	6.94961	6.18991	0.759701	1.69314	Greater than 0
1434249_s_a	94090	Trim9	tripartite mo	NM_001110	0.0313854	0.680144	4.97624	8.04332	7.1751	0.868221	1.82541	Greater than 0
1457111_at	103570	AA415038	expressed se	---	0.0316847	0.68113	3.23967	7.83945	7.20433	0.635112	1.55306	Greater than 0
1455050_at	320736	E130203B14	RIKEN cDNA	NM_178791	0.0349747	0.692791	4.91004	6.87443	6.01602	0.858414	1.81304	Greater than 0
1422789_at	19378	Aldh1a2	aldehyde del	NM_009022	0.0365687	0.701318	3.13756	6.34446	5.54955	0.794904	1.73496	Greater than 0
1428738_a_a	100039192 /	D14Ertd449e	DNA segmen	NM_025311	0.0374852	0.70729	3.93042	8.80065	8.06658	0.734076	1.66333	Greater than 0
1418937_at	13371	Dio2	deiodinase, i	NM_010050	0.037696	0.709469	4.79264	7.53049	6.85241	0.678076	1.60001	Greater than 0
1422230_s_a	13086 /// 13	Cyp2a4 /// C	cytochrome	NM_007812	0.0425058	0.718655	2.93842	4.55088	3.94786	0.603025	1.5189	Greater than 0
1439143_at	328399	A930018M24	RIKEN cDNA	XR_141243 /	0.04317	0.719272	2.92299	5.85523	5.19769	0.657543	1.57739	Greater than 0
