# Supplementary material for: The Nuclear Receptor REV-ERBα Regulates Fabp7 and Modulates Adult Hippocampal Neurogenesis
Source: PLoS One. 2014 Jun 16;9(6):e99883. doi: 10.1371/journal.pone.0099883 (PMC4059695; doi:10.1371/journal.pone.0099883)
Supplement: Table S2 — List of genes down-regulated in Rev-erbα KO SCN. (PDF) [file pone.0099883.s006.pdf]

Table S2

Probeset ID	Entrez Gene	Gene Symbol	Gene Title	RefSeq Transcript ID	p-value	stepup(p-value)	t	Mean(Rev Erb (--))	Mean(WT)	MeanDiff(Rev Erb (--)-WT)	FoldChange(Rev Erb (--)/WT)	FoldChange(Rev Erb (--)/WT)(Description)
1454675_at	21833	Thra	thyroid horm	NM_178060	8.29E-06	0.145299	-57.5004	10.3989	11.537	-1.13814	-2.20097	Less than 0
1428563_at	77591	Ddx10	DEAD (Asp-G	NM_029936	9.66E-06	0.145299	-40.076	6.26929	6.99888	-0.729584	-1.65816	Less than 0
1440282_at	68842	Tulp4	tubby like pr	NM_001033	1.81E-05	0.172295	-23.9887	4.68877	5.96144	-1.27267	-2.41608	Less than 0
1438565_at	231570	A830010M20	RIKEN cDNA	NM_001007	2.50E-05	0.188073	-26.9724	6.54156	7.6672	-1.12564	-2.18198	Less than 0
1438207_at	107338	Gbf1	golgi-specific	NM_178930	4.64E-05	0.298818	-19.2931	5.8974	7.52782	-1.63042	-3.09602	Less than 0
1450051_at	22589	Atrx	alpha thalass	NM_009530	5.65E-05	0.318414	-22.9907	6.72001	7.88493	-1.16492	-2.24221	Less than 0
1421064_at	56217	Mpp5	membrane p	NM_019579	9.18E-05	0.343755	-16.3982	8.53567	9.35092	-0.815258	-1.75961	Less than 0
1425600_a_a	18795	Plcb1	phospholipas	NM_001145	0.00010242	0.343755	-15.6636	6.61235	7.70958	-1.09723	-2.13943	Less than 0
1429592_at	269629	Lhfpl3	lipoma HMG	NM_001081	0.00010583	0.343755	-28.8713	6.77337	7.66255	-0.889185	-1.85213	Less than 0
1429362_a_a	319322	Sf3b2	splicing facto	NM_030109	0.00012957	0.343755	-14.6097	9.77683	10.7194	-0.942587	-1.92197	Less than 0
1431191_a_a	20979	Syt1	synaptotagm	NM_001252	0.00016482	0.371673	-15.1937	8.11886	8.98122	-0.862362	-1.81801	Less than 0
1426464_at	217166	Nr1d1	nuclear recep	NM_145434	0.00026119	0.40141	-16.6078	5.6937	7.53226	-1.83856	-3.57653	Less than 0
1421592_at	17968	Ncam2	neural cell ad	NM_001113	0.00026185	0.40141	-14.1617	6.9476	7.61969	-0.672097	-1.59339	Less than 0
1453860_s_a	14815	Nr3c1	nuclear recep	NM_008173	0.00028481	0.40141	-15.7622	5.30114	6.22606	-0.924923	-1.89858	Less than 0
1453735_at	70591	5730455P16	RIKEN cDNA	NM_027472	0.00034631	0.417158	-11.5992	7.00214	7.66978	-0.667648	-1.58848	Less than 0
1438040_a_a	22027	Hsp90b1	heat shock p	NM_011631	0.00037194	0.417158	-19.7865	9.08621	9.72152	-0.635318	-1.55328	Less than 0
1437657_at	244891	Scaper	S phase cycli	NM_001081	0.00042978	0.417158	-10.7366	7.04936	8.05105	-1.00169	-2.00234	Less than 0
1428574_a_a	69993	Chn2	chimerin (chi	NM_001163	0.00043203	0.417158	-17.9418	6.31732	7.14272	-0.825401	-1.77203	Less than 0
1435770_at	52837	Tmx4	thioredoxin-r	NM_029148	0.00051243	0.417158	-10.2443	7.37744	8.87368	-1.49625	-2.82108	Less than 0
1436850_at	263764	Creg2	cellular repre	NM_170597	0.00054542	0.417158	-10.3106	6.73221	7.56694	-0.834733	-1.78353	Less than 0
1456112_at	108989	Tpr	translocated	NM_133780	0.00055579	0.417158	-11.1511	8.48123	9.32965	-0.848423	-1.80053	Less than 0
1424598_at	13209	Ddx6	DEAD (Asp-G	NM_001110	0.00058353	0.417158	-11.0825	8.79573	9.81184	-1.01611	-2.02246	Less than 0
1426444_at	215160	Rhbdd2	rhomboid do	NM_031398	0.00060305	0.417158	-11.6061	8.03791	8.6863	-0.648393	-1.56742	Less than 0
1416062_at	66687	Tbc1d15	TBC1 domain	NM_025706	0.0006314	0.417158	-9.73818	5.73914	6.38127	-0.642122	-1.56062	Less than 0
1452360_a_a	214899	Kdm5a	lysine (K)-spe	NM_145997	0.00063605	0.417158	-10.0428	8.0063	8.76201	-0.75571	-1.68846	Less than 0
1459563_x_a	---	---	---	---	0.00067641	0.417158	-10.7449	4.73154	5.36093	-0.62939	-1.54691	Less than 0
1453787_at	52837	Tmx4	thioredoxin-r	NM_029148	0.00070536	0.417158	-13.3261	7.36139	8.72698	-1.36559	-2.57682	Less than 0
1429660_s_a	14211	Smc2	structural ma	NM_008017	0.00071179	0.417158	-9.4094	3.70653	4.40251	-0.695988	-1.61999	Less than 0
1446914_at	96999	C80425	expressed se	---	0.00071911	0.417158	-9.80337	4.28355	5.1542	-0.870659	-1.8285	Less than 0

1455151_at	100986	Akap9	A kinase (PRI	NM_194462	0.00082171	0.417158	-16.9228	6.88396	7.56801	-0.684054	-1.60665	Less than 0
1416482_at	22129	Ttc3	tetratricopep	NM_009441	0.00083769	0.417158	-9.07615	9.67229	10.7011	-1.02885	-2.04039	Less than 0
1420973_at	71371	Arid5b	AT rich intera	NM_023598	0.00085473	0.417158	-9.0334	6.78029	7.37206	-0.591762	-1.50709	Less than 0
1424207_at	93762	Smarca5	SWI/SNF rela	NM_053124	0.00088795	0.417158	-14.8229	8.19611	8.97151	-0.775399	-1.71166	Less than 0
1450915_at	11774	Ap3b1	adaptor-relat	NM_009680	0.00092129	0.42212	-8.79862	7.32067	8.13032	-0.809649	-1.75278	Less than 0
1452236_at	224742	Abcf1	ATP-binding	NM_013854	0.00094044	0.42212	-12.4387	8.26334	8.85783	-0.594496	-1.50995	Less than 0
1439300_at	12212	Chic1	cysteine-rich	NM_009767	0.00096272	0.42212	-9.26171	8.03765	8.80972	-0.772066	-1.70771	Less than 0
1419038_a_a	12995	Csnk2a1	casein kinase	NM_007788	0.00096402	0.42212	-10.832	7.2935	8.02841	-0.734907	-1.66429	Less than 0
1430511_at	208898	Unc13c	unc-13 homo	NM_001081	0.00098904	0.424827	-8.81433	4.99692	6.35374	-1.35682	-2.56121	Less than 0
1436343_at	107932	Chd4	chromodoma	NM_145979	0.00102706	0.436995	-25.0867	8.57392	9.41203	-0.838113	-1.78771	Less than 0
1425019_at	217379	Ubxn2a	UBX domain	NM_145441	0.00104383	0.439979	-8.6101	8.67635	9.27266	-0.596317	-1.51185	Less than 0
1452788_at	26932	Ppp2r5e	protein phos	NM_012024	0.00114816	0.448151	-12.1307	8.4145	9.02585	-0.611348	-1.52769	Less than 0
1460304_a_a	21429	Ubtf	upstream bir	NM_001044	0.00116304	0.448151	-8.46376	5.66177	6.64695	-0.985184	-1.97957	Less than 0
1429432_at	226562	Prrc2c	proline-rich c	NM_001081	0.00123428	0.448151	-8.68462	3.4898	4.41393	-0.924135	-1.89755	Less than 0
1422842_at	24128	Xrn2	5'-3' exoribo	NM_011917	0.00124464	0.448151	-8.1725	6.85421	7.64615	-0.791943	-1.7314	Less than 0
1453263_at	78689	Naa35	N(alpha)-ace	NM_030153	0.00124631	0.448151	-9.11406	7.49804	8.16073	-0.662687	-1.58303	Less than 0
1456489_at	74737	Pcf11	cleavage and	NM_029078	0.00132201	0.448151	-9.52918	6.26184	7.00035	-0.738513	-1.66846	Less than 0
1438975_x_a	224454	Zdhhc14	zinc finger, D	NM_146073	0.00133523	0.448151	-8.12341	7.91915	8.66103	-0.741878	-1.67235	Less than 0
1458385_at	18415	Hspa4l	heat shock p	NM_011020	0.00135242	0.448151	-9.67776	7.70508	8.32114	-0.616067	-1.53269	Less than 0
1437614_x_a	224454	Zdhhc14	zinc finger, D	NM_146073	0.00137688	0.448151	-8.39227	7.8059	8.97614	-1.17024	-2.25049	Less than 0
1417831_at	24061	Smc1a	structural ma	NM_019710	0.00137693	0.448151	-10.8343	7.56789	8.49408	-0.926186	-1.90025	Less than 0
1456088_at	11798	Xiap	X-linked inhil	NM_009688	0.00144412	0.448151	-11.4223	7.08267	8.08023	-0.997559	-1.99662	Less than 0
1424922_a_a	57261	Brd4	bromodomai	NM_020508	0.00147872	0.448151	-9.36023	8.78371	9.46619	-0.682479	-1.60489	Less than 0
1430568_at	67302	Zc3h13	zinc finger C(	NM_026083	0.00150258	0.448151	-8.65974	7.0321	7.89956	-0.867464	-1.82445	Less than 0
1428046_a_a	22764	Zfx	zinc finger pr	NM_001044	0.00160795	0.450394	-8.59517	4.3965	5.39484	-0.998343	-1.9977	Less than 0
1456110_at	77087	Ankrd11	ankyrin repe	NM_001081	0.0016364	0.450394	-7.57194	7.90871	8.77301	-0.8643	-1.82046	Less than 0
1452885_at	72193	Scaf11	SR-related CT	NM_028148	0.00163776	0.450394	-9.22071	7.11032	8.14832	-1.038	-2.05337	Less than 0
1426485_at	67812	Ubxn4	UBX domain	NM_026390	0.00175156	0.462159	-9.55496	9.16767	9.81618	-0.64851	-1.56755	Less than 0
1449042_at	13018	Ctcf	CCCTC-bindi	NM_181322	0.00190988	0.463036	-19.9927	8.76159	9.52539	-0.763806	-1.69796	Less than 0
1440248_at	319996	Casc4	cancer susce	NM_001205	0.00192485	0.463036	-13.6699	4.83599	6.36467	-1.52868	-2.88523	Less than 0
1421504_at	20688	Sp4	trans-acting	NM_001166	0.00194249	0.463036	-8.31278	7.00713	7.59429	-0.58716	-1.50229	Less than 0
1456874_at	399558	Flrt2	fibronectin le	NM_201518	0.00194439	0.463036	-7.78557	5.80015	6.51149	-0.711338	-1.63732	Less than 0
1459295_at	---	---	---	---	0.00197836	0.463036	-7.20852	5.34683	6.64959	-1.30276	-2.46701	Less than 0
1415859_at	56347	Eif3c	eukaryotic tr	NM_146200	0.00198584	0.463036	-12.5396	10.0884	10.7141	-0.625675	-1.54293	Less than 0

1439122_at	13209	Ddx6	DEAD (Asp-G	NM_001110	0.00198967	0.463036	-13.4326	8.11224	8.8254	-0.713164	-1.6394	Less than 0
1420989_at	66756	4933411K20	RIKEN cDNA	NM_025747	0.00203649	0.466232	-8.0946	5.82198	7.2256	-1.40362	-2.64564	Less than 0
1431465_s_a	69823	Fyttd1	forty-two-th	NM_001159	0.00209654	0.474935	-7.08423	5.76831	6.3973	-0.62899	-1.54648	Less than 0
1424675_at	106957	Slc39a6	solute carrie	NM_139143	0.0023415	0.48237	-7.37129	7.37571	8.0029	-0.627189	-1.54455	Less than 0
1457281_at	78244	Dnajc21	DnaJ (Hsp40)	NM_030046	0.0024344	0.488897	-12.1758	7.34354	8.07158	-0.728036	-1.65638	Less than 0
1456316_a_a	170760	Acbd3	acyl-Coenzym	NM_133225	0.00247563	0.488897	-12.4236	6.37963	7.14466	-0.76503	-1.69941	Less than 0
1431686_a_a	63985	Gmfb	glia maturati	NM_022023	0.0024941	0.488897	-9.34869	5.20928	5.83166	-0.62238	-1.53941	Less than 0
1438101_at	14365	Fzd3	frizzled homo	NM_021458	0.00257453	0.493072	-6.7163	3.75187	4.39572	-0.643857	-1.5625	Less than 0
1427822_a_a	100044236	Copg2as2	coatomer pro	NR_002845	0.00259105	0.493072	-13.2608	7.33528	8.65704	-1.32177	-2.49972	Less than 0
1438363_at	434128	Pnmal2	PNMA-like 2	NM_001099	0.0026402	0.493072	-12.9838	9.72514	10.9654	-1.24029	-2.36245	Less than 0
1460384_a_a	94246	Arid4b	AT rich intera	NM_194262	0.00272016	0.496688	-6.7312	5.63002	6.34373	-0.713707	-1.64001	Less than 0
1424142_at	230233	Ikbkap	inhibitor of k	NM_026079	0.00281524	0.503523	-7.25351	6.69231	7.43614	-0.743824	-1.67461	Less than 0
1438476_a_a	107932	Chd4	chromodoma	NM_145979	0.00287545	0.503523	-7.11465	7.09708	7.92828	-0.831197	-1.77916	Less than 0
1420402_at	11941	Atp2b2	ATPase, Ca++	NM_001036	0.00294107	0.503523	-9.90205	7.55284	8.76207	-1.20923	-2.31214	Less than 0
1426541_a_a	71946	Endod1	endonucleas	NM_028013	0.00302446	0.503523	-6.42443	7.83822	8.46688	-0.628662	-1.54613	Less than 0
1458539_at	226412	R3hdm1	R3H domain	NM_181750	0.00306733	0.503523	-6.46901	5.53213	6.48842	-0.956295	-1.94032	Less than 0
1424325_at	77805	Esco1	establishmen	NM_001081	0.00309521	0.503961	-6.38801	6.52643	7.44576	-0.919327	-1.89123	Less than 0
1436229_at	213056	Fam126b	family with s	NM_172513	0.00319798	0.50788	-8.41685	8.51144	9.31407	-0.80263	-1.74428	Less than 0
1455905_at	72503	2610507B11	RIKEN cDNA	NM_001002	0.00321067	0.50788	-7.98834	7.82022	8.6391	-0.818884	-1.76404	Less than 0
1424658_at	216965	Taok1	TAO kinase 1	NM_144825	0.0032724	0.50788	-7.91845	6.54172	7.42605	-0.884327	-1.8459	Less than 0
1445081_at	320271	Scai	suppressor o	NM_178778	0.00328951	0.50788	-6.28049	6.03919	6.88654	-0.84736	-1.79921	Less than 0
1447869_x_a	73296	Rhobtb3	Rho-related I	NM_028493	0.00331632	0.50788	-7.14423	6.61191	7.36241	-0.750494	-1.68237	Less than 0
1438774_s_a	70974	Pgm2l1	phosphogluc	NM_027629	0.00336711	0.50788	-6.60791	8.5799	9.20057	-0.620671	-1.53759	Less than 0
1450093_s_a	16969	Zbtb7a	zinc finger ar	NM_010731	0.00356412	0.50788	-7.16159	7.14616	7.93982	-0.793659	-1.73346	Less than 0
1439555_at	109263	Rlf	rearranged L	NM_001081	0.00357172	0.50788	-16.0932	3.92951	5.6067	-1.67719	-3.19804	Less than 0
1445296_at	52480	D7Ertd715e	DNA segmen	NR_015456	0.00357992	0.50788	-6.2761	6.22705	6.98805	-0.761	-1.69466	Less than 0
1418129_at	74754	Dhcr24	24-dehydroc	NM_053272	0.00362852	0.50788	-6.63792	8.20124	8.83393	-0.632696	-1.55046	Less than 0
1442100_at	101490	Inpp5f	inositol polyp	NM_178641	0.00367506	0.50788	-6.10057	5.44492	6.24469	-0.799773	-1.74083	Less than 0
1446512_at	69082	Zc3h15	zinc finger C	NM_026934	0.00368153	0.50788	-6.66769	5.75852	6.55634	-0.797817	-1.73847	Less than 0
1422042_at	118446	Gjc3	gap junction	NM_080450	0.00376243	0.50788	-6.22028	5.81131	6.40237	-0.591063	-1.50636	Less than 0
1456337_at	212285	Arap2	ArfGAP with	NM_178407	0.00379225	0.50788	-6.34152	3.62451	4.51998	-0.895466	-1.86021	Less than 0
1452479_at	100044236	Copg2as2	coatomer pro	NR_002845	0.00385484	0.50788	-6.63357	3.82631	4.93464	-1.10833	-2.15596	Less than 0
1422675_at	57376	Smarce1	SWI/SNF rela	NM_020618	0.00391119	0.508353	-9.71176	6.90334	7.51276	-0.609421	-1.52565	Less than 0
1431024_a_a	94246	Arid4b	AT rich intera	NM_194262	0.00408997	0.509084	-7.88852	4.73476	5.67892	-0.944156	-1.92406	Less than 0

1451878_a_a	57748	Jmy	junction-med	NM_021310	0.00411955	0.509084	-5.90678	3.53546	4.94513	-1.40967	-2.65676	Less than 0
1422834_at	16508	Kcnd2	potassium vo	NM_019697	0.00425371	0.510357	-14.8901	8.47153	9.28639	-0.814856	-1.75912	Less than 0
1439642_at	320504	5930403N24	RIKEN cDNA	NM_177177	0.00436413	0.511218	-7.02627	4.44526	5.44475	-0.999489	-1.99929	Less than 0
1437107_at	270192	Rab6b	RAB6B, mem	NM_173781	0.00437118	0.511218	-10.0886	8.9613	10.0404	-1.07911	-2.11273	Less than 0
1419250_a_a	18647	Cdk14	cyclin-depen	NM_011074	0.00437396	0.511218	-10.569	6.6017	7.29958	-0.697873	-1.62211	Less than 0
1447723_at	---	---	---	---	0.00440419	0.511218	-5.83346	6.43667	7.50909	-1.07242	-2.10296	Less than 0
1432558_a_a	17153	Mal	myelin and ly	NM_001171	0.00453858	0.511218	-7.1478	8.78276	9.42077	-0.638005	-1.55618	Less than 0
1458147_at	---	---	---	---	0.00459869	0.511218	-6.20779	5.40435	6.32042	-0.916073	-1.88697	Less than 0
1422910_s_a	67241	Smc6	structural ma	NM_025695	0.00466635	0.51331	-6.21598	8.22115	8.9026	-0.68145	-1.60375	Less than 0
1426892_at	22288	Utrn	utrophin	NM_011682	0.00485691	0.513394	-6.09883	4.65364	5.51313	-0.859487	-1.81439	Less than 0
1425544_at	109135	Plekha5	pleckstrin ho	NM_144920	0.00491491	0.513394	-6.53273	5.33528	5.92717	-0.59189	-1.50722	Less than 0
1419092_a_a	20874	Slk	STE20-like ki	NM_001164	0.0049221	0.513394	-13.1611	4.76434	5.56568	-0.801338	-1.74272	Less than 0
1428092_at	71702	Cdc5l	cell division c	NM_152810	0.00512478	0.52282	-7.95767	8.10234	8.7073	-0.604961	-1.52094	Less than 0
1460426_at	83679	Pde4dip	phosphodies	NM_001039	0.0052398	0.52282	-9.97689	6.0732	6.89936	-0.826165	-1.77297	Less than 0
1422206_at	26877	B3galt1	UDP-Gal:beta	NM_020283	0.0052437	0.52282	-6.29826	5.43803	6.15824	-0.720211	-1.64742	Less than 0
1438719_at	26405	Map3k2	mitogen-acti	NM_011946	0.00524619	0.52282	-9.58466	5.08427	5.82803	-0.743762	-1.67454	Less than 0
1417623_at	20496	Slc12a2	solute carrier	NM_009194	0.00563322	0.52282	-8.81011	7.68833	8.43884	-0.750508	-1.68239	Less than 0
1456656_at	108030	Lin7a	lin-7 homolo	NM_001033	0.00564294	0.52282	-5.42459	7.48631	8.0743	-0.587992	-1.50315	Less than 0
1420947_at	22589	Atrx	alpha thalass	NM_009530	0.00570078	0.52282	-6.53787	6.42074	7.27009	-0.84935	-1.80169	Less than 0
1427510_at	14677	Gnai1	guanine nucl	NM_010305	0.0058313	0.52282	-11.4799	7.37993	8.00942	-0.629491	-1.54702	Less than 0
1439083_at	52906	Ahi1	Abelson help	NM_001177	0.00583553	0.52282	-5.88906	8.82504	9.4386	-0.61356	-1.53003	Less than 0
1456187_at	241919	Slc7a14	solute carrier	NM_172861	0.00583912	0.52282	-7.05912	5.2592	6.2321	-0.972897	-1.96278	Less than 0
1426168_a_a	100038850	Trav9d-3	T cell recepto	---	0.00586904	0.524055	-5.49727	5.21506	5.93242	-0.717361	-1.64417	Less than 0
1453855_at	67622	Mxra7	matrix-remod	NM_026280	0.00617814	0.53109	-5.44441	5.40823	6.2429	-0.834666	-1.78344	Less than 0
1429308_at	70673	Prdm16	PR domain co	NM_001177	0.00618217	0.53109	-5.67118	4.99135	6.06568	-1.07433	-2.10575	Less than 0
1440623_at	233919	Gpr26	G protein-co	NM_173410	0.00671735	0.541051	-9.31403	4.98377	5.64916	-0.665388	-1.586	Less than 0
1457040_at	246316	Lgi2	leucine-rich r	NM_144945	0.00692075	0.545117	-5.36202	6.50926	7.22928	-0.720024	-1.64721	Less than 0
1430534_at	78416	Rnase6	ribonuclease	NM_030098	0.00693693	0.545117	-5.28748	4.57249	5.2867	-0.714212	-1.64059	Less than 0
1422546_at	16201	Ilf3	interleukin e	NM_001042	0.00697275	0.545331	-7.17232	6.5062	7.23389	-0.727687	-1.65598	Less than 0
1450530_at	26877	B3galt1	UDP-Gal:beta	NM_020283	0.007003	0.546405	-9.42999	4.62763	5.35606	-0.728429	-1.65683	Less than 0
1421905_at	116940	Tgs1	trimethylgua	NM_054089	0.00708355	0.547985	-10.1173	7.10666	7.69211	-0.585455	-1.50051	Less than 0
1459804_at	12914	Crebbp	CREB binding	NM_001025	0.00709576	0.54799	-7.72273	5.07703	6.53952	-1.4625	-2.75585	Less than 0
1418431_at	16573	Kif5b	kinesin famil	NM_008448	0.00717349	0.548256	-9.13812	8.71626	9.38258	-0.666314	-1.58701	Less than 0
1451800_at	70297	Gcc2	GRIP and coi	NM_027375	0.00745448	0.55235	-5.34644	5.17199	5.79678	-0.624789	-1.54199	Less than 0

1420911_a_a	17304	Mfge8	milk fat globu	NM_0010454	0.00746937	0.55235	-5.2189	9.87513	10.5682	-0.693097	-1.61675	Less than 0
1439605_at	---	---	---	---	0.00748511	0.55235	-7.55402	6.69513	7.46869	-0.773561	-1.70948	Less than 0
1432269_a_a	58194	Sh3kbp1	SH3-domain	NM_0011357	0.00757431	0.55235	-8.28449	6.40513	7.23002	-0.824888	-1.7714	Less than 0
1427456_at	72145	Wdfy3	WD repeat a	NM_172882	0.00766101	0.55235	-6.2328	8.0485	8.67682	-0.628316	-1.54576	Less than 0
1420946_at	22589	Atrx	alpha thalass	NM_009530	0.00767979	0.55235	-4.97792	5.87877	6.82167	-0.942908	-1.9224	Less than 0
1456863_at	13838	Epha4	Eph receptor	NM_007936	0.00769604	0.55235	-7.19441	5.35901	5.97871	-0.619699	-1.53655	Less than 0
1439566_at	243385	Gprin3	GPRIN family	NM_183183	0.00769622	0.55235	-5.38938	5.09499	5.94404	-0.849055	-1.80132	Less than 0
1427037_at	208643	Eif4g1	eukaryotic tr	NM_0010053	0.00770086	0.55235	-6.97938	6.32363	7.13381	-0.810184	-1.75343	Less than 0
1449939_s_a	13386	Dlk1	delta-like 1 h	NM_0011907	0.00788477	0.554117	-8.60547	9.03117	9.71637	-0.6852	-1.60792	Less than 0
1420935_a_a	51796	Srrm1	serine/argini	NM_0011304	0.00806011	0.558231	-5.517	8.3401	8.92831	-0.588207	-1.50338	Less than 0
1420917_at	56194	Prpf40a	PRP40 pre-m	NM_018785	0.00816977	0.558231	-4.94226	5.92265	6.66198	-0.739329	-1.6694	Less than 0
1452333_at	67155	Smarca2	SWI/SNF rela	NM_011416	0.00827968	0.562382	-5.12385	6.47483	7.25841	-0.78358	-1.7214	Less than 0
1460729_at	19877	Rock1	Rho-associat	NM_009071	0.00838462	0.564908	-7.97178	7.04196	7.68862	-0.646657	-1.56554	Less than 0
1417832_at	24061	Smc1a	structural ma	NM_019710	0.00851052	0.566962	-6.90593	6.09303	7.04518	-0.952151	-1.93476	Less than 0
1430073_at	74901	Kbtbd11	kelch repeat	NM_029116	0.00853692	0.567881	-6.92592	7.05133	7.95005	-0.898713	-1.8644	Less than 0
1437002_at	215708	Fam73a	family with s	NM_0011623	0.0085738	0.568979	-6.69607	7.91036	8.61954	-0.709183	-1.63488	Less than 0
1450035_a_a	56194	Prpf40a	PRP40 pre-m	NM_018785	0.00864657	0.568979	-7.76323	6.38296	7.13061	-0.747647	-1.67905	Less than 0
1452187_at	83486	Rbm5	RNA binding	NM_148930	0.00866696	0.568979	-4.93903	6.47682	7.21077	-0.733946	-1.66318	Less than 0
1457744_at	212880	Ddx46	DEAD (Asp-G	NM_145975	0.0086971	0.5693	-4.79368	6.63604	7.63842	-1.00238	-2.0033	Less than 0
1427488_a_a	12211	Birc6	baculoviral IA	NM_007566	0.00883164	0.56933	-4.78838	5.4691	6.37124	-0.902139	-1.86884	Less than 0
1437501_at	209743	AF529169	cDNA sequer	NM_153509	0.0088649	0.56933	-4.7689	4.68165	5.37257	-0.690919	-1.61431	Less than 0
1455960_at	230316	Megf9	multiple EGF	NM_172694	0.00944369	0.580595	-5.92213	6.23951	7.56025	-1.32074	-2.49794	Less than 0
1452811_at	108147	Atic	5-aminoimid	NM_026195	0.00952205	0.580746	-9.29372	6.14543	7.05095	-0.905518	-1.87322	Less than 0
1419256_at	20742	Spnb2	spectrin beta	NM_009260	0.00959625	0.580746	-8.16188	10.1395	10.8969	-0.75737	-1.69041	Less than 0
1438801_at	103967	Dnm3	dynamin 3	NM_0010386	0.00963964	0.581074	-4.99337	7.04948	8.06061	-1.01113	-2.01549	Less than 0
1437581_at	627049	Zfp800	zinc finger pr	NM_0010816	0.00968865	0.581074	-7.19728	5.89162	7.16071	-1.26908	-2.41008	Less than 0
1435135_at	320024	Nceh1	arylacetamid	NM_178772	0.00979412	0.585842	-6.9984	9.32894	10.0571	-0.728117	-1.65648	Less than 0
1430974_a_a	66315	Senp7	SUMO1/sent	NM_0010039	0.00995044	0.586241	-4.68303	4.8755	5.7088	-0.833306	-1.78176	Less than 0
1457495_at	73040	2900052N01	RIKEN cDNA	NR_015605	0.0103212	0.586241	-4.79883	6.58452	7.60786	-1.02335	-2.03263	Less than 0
1436311_at	216766	Gemin5	gem (nuclear	NM_0011666	0.0104011	0.586241	-5.8897	3.50192	4.24305	-0.741135	-1.67149	Less than 0
1428047_s_a	22639 /// 22	Zfa /// Zfx	zinc finger pr	NM_0010443	0.0105229	0.586241	-8.94634	5.34648	6.07551	-0.729035	-1.65753	Less than 0
1416661_at	13669	Eif3a	eukaryotic tr	NM_010123	0.0105898	0.586241	-6.88536	6.79167	7.89626	-1.1046	-2.15039	Less than 0
1427311_at	207165	Bptf	bromodomai	NM_0010808	0.0106859	0.586241	-4.77767	4.04456	4.89942	-0.854859	-1.80858	Less than 0
1417362_at	56315	Rhcg	Rhesus blood	NM_019799	0.0109577	0.586241	-7.69601	5.24186	6.16985	-0.92799	-1.90262	Less than 0

1443069_at	---	---	---	---	0.0110223	0.586241	-5.84749	4.98401	5.58184	-0.597827	-1.51344	Less than 0
1449999_a_a	12293	Cacna2d1	calcium chan	NM_001110	0.011024	0.586241	-5.43484	8.39264	9.27768	-0.885034	-1.84681	Less than 0
1450174_at	19281	Ptprt	protein tyros	NM_021464	0.0111498	0.586241	-6.86007	7.14277	7.84864	-0.705874	-1.63113	Less than 0
1421978_at	14417	Gad2	glutamic acid	NM_008078	0.0111714	0.586241	-5.06468	10.177	10.8864	-0.709408	-1.63513	Less than 0
1420837_at	18212	Ntrk2	neurotrophic	NM_001025	0.0113727	0.586241	-5.42674	6.73238	8.05069	-1.31831	-2.49373	Less than 0
1442939_at	51869	Rif1	Rap1 interact	NM_175238	0.0114815	0.586241	-6.72703	6.53987	7.15721	-0.617342	-1.53405	Less than 0
1437554_at	18810	Plec	plectin	NM_001163	0.0116321	0.586241	-5.35934	5.27461	6.00325	-0.728645	-1.65708	Less than 0
1450906_at	54712	Plxnc1	plexin C1	NM_018797	0.011793	0.586241	-7.04445	4.91577	5.92947	-1.0137	-2.01908	Less than 0
1422741_a_a	70508	Bbx	bobby sox ho	NM_027444	0.0118507	0.586241	-4.40056	4.54502	5.37383	-0.828808	-1.77622	Less than 0
1416801_at	58800	Trpm7	transient rec	NM_001164	0.0120784	0.586241	-6.19017	4.94116	5.54481	-0.603642	-1.51955	Less than 0
1454198_a_a	66793	Efcab1	EF hand calci	NM_025769	0.0121762	0.586241	-8.84027	5.9891	6.69529	-0.706192	-1.63149	Less than 0
1457409_at	14348	Fut9	fucosyltransf	NM_010243	0.0123665	0.59057	-5.66063	6.77297	7.44848	-0.675512	-1.59716	Less than 0
1421144_at	77945	Rpgrip1	retinitis pigm	NM_001168	0.0127406	0.595375	-4.88201	8.26697	8.93949	-0.672527	-1.59386	Less than 0
1429665_at	230376	Haus6	HAUS augmin	NM_173400	0.0129826	0.59644	-8.64276	3.4841	4.10664	-0.622539	-1.53958	Less than 0
1440227_at	53881	Slc5a3	solute carrier	NM_017391	0.0133887	0.599436	-4.7066	7.73885	8.33472	-0.595868	-1.51138	Less than 0
1440161_at	17389	Mmp16	matrix metal	NM_019724	0.0139277	0.600797	-4.23021	5.9193	6.71622	-0.796915	-1.73738	Less than 0
1442725_at	---	---	---	---	0.0141133	0.600797	-7.33525	3.49357	4.87854	-1.38497	-2.61166	Less than 0
1455586_at	70238	Rnf168	ring finger pr	NM_027355	0.0141614	0.600797	-5.81486	5.77825	6.48115	-0.702894	-1.62777	Less than 0
1440142_s_a	14580	Gfap	glial fibrillary	NM_001131	0.0143587	0.601418	-6.62807	7.74167	9.10189	-1.36022	-2.56724	Less than 0
1421508_at	23963	Odz1	odd Oz/ten-m	NM_011855	0.0145405	0.601418	-6.31165	7.21185	7.89699	-0.685137	-1.60786	Less than 0
1459310_at	---	---	---	---	0.0147737	0.604636	-4.55227	5.27517	6.43559	-1.16042	-2.23522	Less than 0
1456495_s_a	99031	Osbpl6	oxysterol bin	NM_145525	0.0151501	0.612811	-7.31572	6.0241	6.61814	-0.594038	-1.50947	Less than 0
1417043_at	16816	Lcat	lecithin chole	NM_008490	0.0153651	0.615243	-4.20226	7.2916	7.91594	-0.624341	-1.54151	Less than 0
1434374_at	319604	Fam168a	family with s	NM_178764	0.0154694	0.615243	-5.41012	8.61935	9.237	-0.617648	-1.53437	Less than 0
1450996_at	14308	Fshb	follicle stimu	NM_008045	0.0157356	0.617978	-4.2743	3.02166	3.61035	-0.588689	-1.50388	Less than 0
1449311_at	12013	Bach1	BTB and CNC	NM_007520	0.0157387	0.617978	-5.07024	5.91085	6.62396	-0.713104	-1.63933	Less than 0
1444075_at	70598	Filip1	filamin A inte	NM_001081	0.0160878	0.618833	-5.17225	4.34135	4.9865	-0.645153	-1.5639	Less than 0
1439434_x_a	230863	Sh2d5	SH2 domain c	NM_001099	0.0162394	0.618833	-6.74252	5.09326	5.72788	-0.634628	-1.55254	Less than 0
1448885_at	74012	Rap2b	RAP2B, mem	NM_028712	0.0164158	0.618833	-5.77843	7.34396	7.93919	-0.59523	-1.51071	Less than 0
1457603_at	243725	Ppp1r9a	protein phos	NM_181595	0.0165274	0.618833	-5.19585	6.03874	6.77735	-0.738615	-1.66857	Less than 0
1456791_at	627049	Zfp800	zinc finger pr	NM_001081	0.0165338	0.618833	-7.30734	6.46716	7.23342	-0.766264	-1.70086	Less than 0
1421955_a_a	17999	Nedd4	neural precu	NM_010890	0.0165676	0.618833	-6.25216	8.49727	10.1927	-1.69544	-3.23875	Less than 0
1438271_at	210126	Lpp	LIM domain c	NM_001145	0.0166886	0.618833	-5.18268	4.41781	5.30194	-0.884124	-1.84564	Less than 0
1417561_at	11812	Apoc1	apolipoprote	NM_001110	0.0168647	0.618833	-5.18061	8.74078	9.39048	-0.649703	-1.56885	Less than 0

1456807_at	105651	Ppp1r3e	protein phos	NM_0011679	0.0175712	0.622453	-5.30695	5.20124	5.95676	-0.755512	-1.68823	Less than 0
1436238_at	213469	Lgi3	leucine-rich r	NM_145219	0.0176585	0.622453	-4.13521	6.67184	7.28915	-0.617306	-1.53401	Less than 0
1424077_at	66569	Gdpd1	glycerophosp	NM_025638	0.0178287	0.622453	-4.69849	7.2175	8.39461	-1.17711	-2.26124	Less than 0
1458050_at	---	---	---	---	0.0182373	0.627674	-3.89998	5.25558	5.92255	-0.666964	-1.58773	Less than 0
1454655_at	227333	Dgkd	diacylglycero	NM_177646	0.0184824	0.628461	-3.85815	5.49193	6.1005	-0.608562	-1.52474	Less than 0
1459377_at	242481	Palm2	paralemmin	NM_172868	0.0185842	0.628951	-4.02842	5.65128	6.25143	-0.600152	-1.51588	Less than 0
1454950_at	319604	Fam168a	family with s	NM_178764	0.018981	0.631049	-5.11581	6.15867	6.80283	-0.644169	-1.56284	Less than 0
1439976_at	207958	Alg11	asparagine-li	NM_0012431	0.0198891	0.632006	-4.61679	5.82114	6.41077	-0.589636	-1.50487	Less than 0
1441667_s_a	12180	Smyd1	SET and MYN	NM_0011601	0.0199174	0.632006	-3.84799	3.3141	3.90397	-0.589875	-1.50512	Less than 0
1442024_at	---	---	---	---	0.020264	0.634286	-6.05598	7.14787	7.83125	-0.683373	-1.60589	Less than 0
1419616_at	12168	Bmpr2	bone morpho	NM_007561	0.0206284	0.634682	-5.71297	5.84677	6.66288	-0.816112	-1.76065	Less than 0
1453391_at	74062 /// 75	Speer7-ps1 /	spermatogen	NR_001584 /	0.0209128	0.634794	-3.72075	5.60597	6.50659	-0.900622	-1.86687	Less than 0
1429517_at	78287	Zfyve20	zinc finger, F	NM_030081	0.0214375	0.63903	-5.70172	4.95755	5.82043	-0.86288	-1.81867	Less than 0
1460476_s_a	71721	Fam13c	family with s	NM_0011437	0.0216633	0.642055	-3.68641	5.88521	6.75823	-0.873028	-1.8315	Less than 0
1423325_at	18949	Pnn	pinin	NM_008891	0.0218283	0.644006	-5.65834	5.68755	6.57665	-0.889108	-1.85203	Less than 0
1442038_at	74213	Rbm26	RNA binding	NM_134077	0.0220137	0.644813	-5.69447	7.94227	8.56153	-0.619265	-1.53609	Less than 0
1447231_at	---	---	---	---	0.0224728	0.648045	-4.19665	5.35242	6.25644	-0.904019	-1.87127	Less than 0
1437018_at	239157	Pnma2	paraneoplast	NM_175498	0.023543	0.648545	-4.55529	6.48872	7.65519	-1.16647	-2.24461	Less than 0
1456157_at	---	---	---	---	0.0237301	0.648545	-3.56409	3.8187	4.50561	-0.686912	-1.60983	Less than 0
1422048_at	22067	Trpc5	transient rec	NM_009428	0.0241909	0.650232	-5.71955	6.41887	7.09509	-0.676222	-1.59795	Less than 0
1436023_at	72567	Bclaf1	BCL2-associa	NM_0010253	0.024259	0.650232	-5.95873	7.64728	8.72593	-1.07866	-2.11207	Less than 0
1450068_at	22385	Baz1b	bromodomai	NM_011714	0.0265258	0.658415	-5.36667	5.23986	6.33293	-1.09306	-2.13327	Less than 0
1456255_at	230249	AI314180	expressed se	NM_172381	0.0268833	0.661661	-4.14122	3.77836	4.63477	-0.856414	-1.81053	Less than 0
1429993_s_a	100039045 /	Gm10471 ///	predicted ge	NM_0011775	0.0269137	0.661661	-5.02839	5.15509	5.82136	-0.666271	-1.58697	Less than 0
1437640_at	230235	6430704M03	RIKEN cDNA	NM_0011429	0.0286052	0.670146	-3.99982	9.35075	9.9613	-0.610556	-1.52685	Less than 0
1428045_a_a	69257	Elf2	E74-like facto	NM_023502	0.0302199	0.677988	-5.33881	5.36074	6.07398	-0.713235	-1.63948	Less than 0
1438732_at	100042480	Nhsl2	NHS-like 2	NM_0011636	0.0303407	0.678713	-5.32603	4.84084	6.32883	-1.48799	-2.80499	Less than 0
1434585_at	68842	Tulp4	tubby like pro	NM_0010335	0.0307171	0.680144	-5.25922	6.44599	7.09093	-0.644934	-1.56367	Less than 0
1428414_at	67974	Ccny	cyclin Y	NM_026484	0.031426	0.680144	-3.86866	4.36941	5.03044	-0.66103	-1.58121	Less than 0
1441797_at	---	---	---	---	0.0327735	0.685265	-3.92113	6.09683	6.69234	-0.595513	-1.51101	Less than 0
1445673_at	73040	2900052N01	RIKEN cDNA	NR_015605	0.0334452	0.688192	-3.39454	6.74268	7.76178	-1.0191	-2.02665	Less than 0
1436231_at	73040	2900052N01	RIKEN cDNA	NR_015605	0.0348509	0.69246	-3.25794	9.83333	10.6341	-0.800767	-1.74203	Less than 0
1459398_at	67245	Peli1	Pellino 1	NM_023324	0.0350544	0.693112	-4.99546	3.53945	4.19991	-0.660455	-1.58058	Less than 0
1418188_a_a	72289	Malat1	metastasis as	NR_002847	0.0352222	0.693996	-3.91717	10.6148	11.559	-0.944205	-1.92413	Less than 0

1440146_at	271564	Vps13a	vacuolar prot	NM_173028	0.0370798	0.705801	-4.5526	4.76762	5.5957	-0.828077	-1.77532	Less than 0
1431047_at	71389	Chd6	chromodoma	NM_173368	0.0378303	0.709764	-4.86198	4.18915	5.19711	-1.00796	-2.01107	Less than 0
1444444_at	---	---	---	---	0.0404362	0.718206	-3.96398	7.42666	8.1242	-0.697541	-1.62174	Less than 0
1446540_at	---	---	---	---	0.0410373	0.71846	-2.9809	4.6769	5.33045	-0.653547	-1.57303	Less than 0
1437118_at	252870	Usp7	ubiquitin spe	NM_0010039	0.0412774	0.71846	-4.68891	5.34181	6.10192	-0.760106	-1.69361	Less than 0
1419277_at	170707	Usp48	ubiquitin spe	NM_130879	0.0413933	0.71846	-3.41877	6.24071	6.92341	-0.682699	-1.60514	Less than 0
1441847_at	---	---	---	---	0.0416694	0.71846	-3.24307	4.07565	4.86386	-0.788207	-1.72693	Less than 0
1444492_at	19266	Ptprd	protein tyros	NM_0010142	0.0418619	0.718655	-3.21276	5.39311	6.68672	-1.29361	-2.45141	Less than 0
1460159_at	320713	Mysm1	myb-like, SW	NM_177239	0.0426826	0.718655	-4.63069	4.66912	5.94816	-1.27904	-2.42678	Less than 0
1460258_at	16840	Lect1	leukocyte cel	NM_010701	0.0428631	0.719062	-3.68551	5.43432	6.0364	-0.602084	-1.51791	Less than 0
1423546_at	22680	Zfp207	zinc finger pr	NM_0011301	0.0431106	0.719272	-2.92395	4.15188	4.8009	-0.649017	-1.5681	Less than 0
1441728_at	20265	Scn1a	sodium chan	NM_018733	0.0431876	0.719272	-2.9832	5.74624	6.47315	-0.726911	-1.65509	Less than 0
1418189_s_a	72289	Malat1	metastasis as	NR_002847	0.0454208	0.721903	-3.83823	11.6871	12.4456	-0.758491	-1.69172	Less than 0
1417645_at	16651	Sspn	sarcospan	NM_010656	0.048296	0.727824	-2.90345	4.52078	5.2152	-0.694416	-1.61823	Less than 0
