# Supplementary material for: The Nuclear Receptor REV-ERBα Regulates Fabp7 and Modulates Adult Hippocampal Neurogenesis
Source: PLoS One. 2014 Jun 16;9(6):e99883. doi: 10.1371/journal.pone.0099883 (PMC4059695; doi:10.1371/journal.pone.0099883)
Supplement: Table S3 — List of oligonucleotides used in this study. (PDF) [file pone.0099883.s007.pdf]

**Table S3**, List of oligonucleotides	
**Primer for ChIP**	**Sequence**
ChIP mFabp7_FW	5’-GGGGATCAGGATTGTGATGT-3’
ChIP mFabp7_RV	5’-AGATGGCTCCAATCCTCCTT-3’
ChIP mFabp7_TM	5’-FAM-TCCGCTAACCCAGTGGCCTGA-BHQ1-3’
ChIP mFgf21_FW	5’-CCATTGCATCATCCGTCCAGGC-3’
ChIP mFgf21_RV	5’-GTGCCCTCCCCACTCCTGAC-3’
ChIP mFgf21_TM	5’-FAM-CGCCCTGGCCACGGTGGAATTCAGG-BHQ1-3’
**Primer for real-time PCR**	**Sequence**
Fabp7 ms_FW	5’-AGCTGGGAGAAGAGTTTGAA-3’
Fabp7 ms_RV	5’-GAGCTTGTCTCCATCCAACC-3’
Fabp7 hs_FW	5’-AAGTCTGTTGTTAGCCTGGA-3’
Fabp7 hs_RV	5’-AGGGTCATAACCATTTTGC-3’
Gapdh_FW	5’-CATGGCCTTCCGTGTTCCTA-3’
Gapdh_RV	5’-CCTGCTTCACCACCTTCTTGA-3’
Rev-erbα_FW	5’-GGGCACAAGCAACATTACCA-3’
Rev-erbα_RV	5’-CACGTCCCCACACACCTTAC-3’
**Primer for cloning of ISH probe**	**Sequence**
Fabp7 ISH_FW	5’-AGACCCGAGTTCCTCCAGTTC-3’
Fabp7 ISH_RV	5’-CCTCCACACCGAAGACAAAC-3’
**Primer for cloning/ mutation of promoter**	**Sequence**
Fabp7 prom_FW	5’-CTGCCTATTTTCAGCTGACTAGGCGGTTAAG-3’
Fabp7 prom_RV	5’-CCATACGTGTGTGCCTTCAAGTCTGAACTAC -3’
Fabp7 promΔRORE_FW	5’-GTGTGAACTGGGAGGATCTGATATCACTCCGCTAACCCAGTGGC-3’
Fabp7 promΔRORE _RV	5’-GCCACTGGGTTAGCGGAGTGATATCAGATCCTCCCAGTTCACAC-3’

FAM: 6-fluorescine; BHQ1: black hole quencher 1
